# Identification and characterisation of spontaneous mutations causing deafness from a targeted knockout programme

**DOI:** 10.1186/s12915-022-01257-8

**Published:** 2022-03-17

**Authors:** Morag A. Lewis, Neil J. Ingham, Jing Chen, Selina Pearson, Francesca Di Domenico, Sohinder Rekhi, Rochelle Allen, Matthew Drake, Annelore Willaert, Victoria Rook, Johanna Pass, Thomas Keane, David J. Adams, Abigail S. Tucker, Jacqueline K. White, Karen P. Steel

**Affiliations:** 1 Wolfson Centre for Age-Related Diseases, King’s College London, London, SE1 1UL England; 2 Wellcome Sanger Institute, Hinxton, CB10 1SA England; 3 Research Group of Experimental Oto-Rhino-Laryngology, Department of Neurosciences, KU Leuven – University of Leuven, Leuven, Belgium; 4 Centre for Craniofacial and Regenerative Biology, King’s College London, London, SE1 9RT England

**Keywords:** Spontaneous mutations, Large-scale mutagenesis programme, Deafness, Progressive hearing loss, Non-segregating phenotypes

## Abstract

**Background:**

Mice carrying targeted mutations are important for investigating gene function and the role of genes in disease, but off-target mutagenic effects associated with the processes of generating targeted alleles, for instance using Crispr, and culturing embryonic stem cells, offer opportunities for spontaneous mutations to arise. Identifying spontaneous mutations relies on the detection of phenotypes segregating independently of targeted alleles, and having a broad estimate of the level of mutations generated by intensive breeding programmes is difficult given that many phenotypes are easy to miss if not specifically looked for. Here we present data from a large, targeted knockout programme in which mice were analysed through a phenotyping pipeline. Such spontaneous mutations segregating within mutant lines may confound phenotypic analyses, highlighting the importance of record-keeping and maintaining correct pedigrees.

**Results:**

Twenty-five lines out of 1311 displayed different deafness phenotypes that did not segregate with the targeted allele. We observed a variety of phenotypes by Auditory Brainstem Response (ABR) and behavioural assessment and isolated eight lines showing early-onset severe progressive hearing loss, later-onset progressive hearing loss, low frequency hearing loss, or complete deafness, with vestibular dysfunction. The causative mutations identified include deletions, insertions, and point mutations, some of which involve new genes not previously associated with deafness while others are new alleles of genes known to underlie hearing loss. Two of the latter show a phenotype much reduced in severity compared to other mutant alleles of the same gene. We investigated the ES cells from which these lines were derived and determined that only one of the 8 mutations could have arisen in the ES cell, and in that case, only after targeting. Instead, most of the non-segregating mutations appear to have occurred during breeding of mutant mice. In one case, the mutation arose within the wildtype colony used for expanding mutant lines.

**Conclusions:**

Our data show that spontaneous mutations with observable effects on phenotype are a common side effect of intensive breeding programmes, including those underlying targeted mutation programmes. Such spontaneous mutations segregating within mutant lines may confound phenotypic analyses, highlighting the importance of record-keeping and maintaining correct pedigrees.

**Supplementary Information:**

The online version contains supplementary material available at 10.1186/s12915-022-01257-8.

## Background

Manipulating embryonic stem (ES) cells to insert or alter DNA to study the effect of targeted genes in the resulting organism is a widely practised technique, and the creation and characterisation of mutant organisms are key steps in exploring gene function. The advent of the CRISPR-Cas9 system has prompted exploration of its potential unwanted off-target mutagenic effects, which are of particular concern for its use as a therapeutic tool. While some studies have reported that off-target effects are “minimal and manageable” [1, 2], with no increase in mutation frequency [2, 3], others have shown that off-target mutations do occur and even alleles present at a low frequency in a G0 mosaic founder could be transmitted to offspring in mice [4]. The creation of targeted mutant alleles involves manipulation of ES cells, and although the mutation frequency is lower in ES cells than in somatic cells [5], mutations do still occur, particularly when the ES cells have been through multiple passages [6]. Spontaneous mutations may thus arise at any point during the process of making a specific allele, including breeding of the resultant organisms.

The Sanger Institute Mouse Genetics Project was a large-scale programme that generated and screened mice carrying knockdown alleles created by the KOMP (Knock Out Mouse Programme) and EUCOMM (European Conditional Mouse Mutagenesis) programmes [7–9]. Each mouse line established carried a single-targeted knockout allele, and animals from each line were put through a wide range of phenotyping tests [9]. One of the tests used was the Auditory Brainstem Response (ABR), a highly sensitive test capable of detecting subtle hearing defects [10, 11]. Vestibular defects leading to balance problems such as circling and head-bobbing were also noted. This enabled the detection of auditory and vestibular phenotypes which did not segregate with the targeted allele and are likely to have been caused by spontaneous mutations. Of the 1311 lines tested, including 2218 wildtype mice and 6798 mutants, we found 25 lines with non-segregating phenotypes. We therefore set out to investigate these spontaneous mutations, firstly in order to better understand how they arose within the Mouse Genetics Project and secondly to identify the genes involved.

## Results

### Identification of spontaneous mutations affecting hearing

**Fig. 1:**
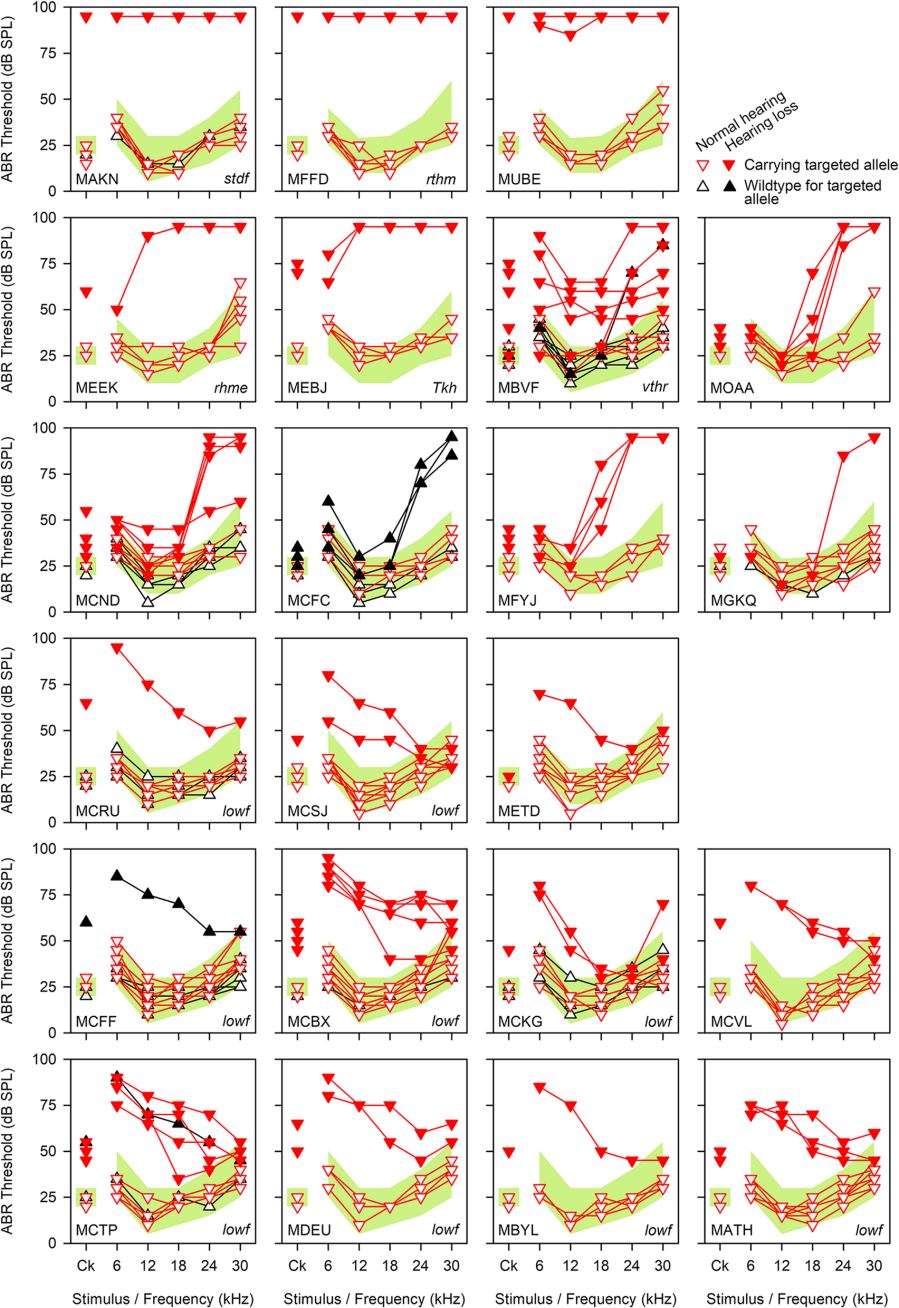
ABR thresholds from 22 targeted lines displaying non-segregating hearing loss. ABR thresholds showing individual mice from targeted mutant lines displaying hearing loss (filled symbols) which does not segregate with the targeted mutant allele (red, inverted triangles) from the original ABR screen. Mice with normal hearing are shown with empty symbols, and black triangles indicate mice which were wildtype for the targeted mutant allele. Affected mice from the MAKN, MFFD and MUBE lines (top) were almost completely deaf. Affected mice from the MEEK and MEBJ colonies exhibited severe hearing loss, while affected mice from the MBVF colony exhibited varying degrees of hearing loss across all frequencies. Affected mice from the MOAA, MCND, MCFC, MFYJ and MGQK colonies (second and third lines) had hearing loss affecting only the higher frequencies, while the remaining eleven colonies all exhibited hearing loss affecting primarily the low frequencies. The shaded pale green area on each panel denotes the 95% reference range for a large population of control wildtype mice derived from littermates of the tested mice, defined in [11]. All mice were tested at 14 weeks old. Mice from three other lines which exhibited non-segregating hearing loss were detected because of their vestibular phenotype (due to the *Tbx1*^*ttch*^, *Pcdh15*^*jigl*^ and *Espn*^*spdz*^ alleles) and did not undergo ABRs as part of the ABR screening pipeline. MAKN *n* = 7 unaffected, 3 affected; MFFD *n* = 5 unaffected, 1 affected; MUBE *n* = 4 unaffected, 2 affected; MEEK *n* = 5 unaffected, 1 affected; MEBJ *n* = 4 unaffected, 2 affected; MBVF *n* = 11 unaffected, 6 affected; MOAA *n* = 3 unaffected, 6 affected; MCND *n* = 9 unaffected, 5 affected; MCFC *n* = 7 unaffected, 3 affected; MFYJ *n* = 3 unaffected, 4 affected; MGKQ *n* = 9 unaffected, 1 affected; MCRU *n* = 11 unaffected, 1 affected; MCSJ *n* = 7 unaffected, 2 affected; METD *n* = 7 unaffected, 1 affected; MDEU *n* = 5 unaffected, 2 affected; MCFF *n* = 16 unaffected, 1 affected; MCBX *n* = 9 unaffected, 5 affected; MCKG *n* = 8 unaffected, 2 affected; MCVL *n* = 7 unaffected, 2 affected; MCTP *n* = 7 unaffected, 5 affected; MBYL *n* = 5 unaffected, 1 affected; MATH *n* = 8 unaffected, 3 affected. Data underlying these plots are in Additional File 3

From our routine ABR screening of mice carrying targeted knockout alleles, we identified twenty-two lines where a hearing impairment phenotype did not segregate with the targeted allele (Fig. 1, Table 1). An additional four lines were discovered because they displayed a vestibular defect, one of which (MFFD) had also been detected through the ABR screen, making twenty-five lines in total. In this study, a mouse line or colony refers to mice descended from a single-mouse carrying a targeted knockout allele bred to a wildtype mouse of the same background. Line or colony names (MXXX) were arbitrarily assigned to each breeding colony to use as part of the unique identifier of each mouse and refer to mice carrying both mutant and wildtype alleles within each colony. After each mouse with an aberrant phenotype was discovered, we screened closely related mice from the same colony and were able to obtain eight mutant lines displaying reliable inheritance of the observed phenotype (Table 1). We used a standard positional cloning approach to identify the mutations. We set up backcrosses to identify the critical chromosomal region for each mutation (Additional File 1: Fig. S1) and carried out whole exome sequencing to identify candidate mutations within each region. We resequenced candidate variants by Sanger sequencing and tested the segregation of the candidate mutation with the phenotype. For two mutations, we confirmed causation using a complementation test. We were able to identify the causative mutation in all eight lines (Table 1), one of which, *S1pr2*^*stdf*^, has already been described [12]. We then confirmed the presence of one of the mutations in 8 more lines, all displaying the same unusual phenotype. Of the 25 lines with aberrant phenotypes, we have identified the causative mutation in 16 of them.

**Table 1:** Summary of the 25 lines found to have a spontaneous mutation affecting hearing

Allele name	Allele symbol	Targeted gene	Line	Phenotype	Mutation	Affected gene(s)	MGI Allele symbol	MGI ID	Mutation type	Mutation effect	Inheri-tance	Parental ES Cell line	Mutation present in parental ES cell line	Mutation type compared to other reported mutant alleles in gene
Stone deaf	*stdf*	*Mms22l*	MAKN	Rapidly progressive severe hearing loss	g.9:20967665G > C	*S1pr2*	*S1pr2* < *stdf* >	MGI:5423977	Missense	T289R	Recessive	JM8.N4	No	Similar
Tikho	*Tkh*	*Rasal2*	MEBJ	Progressive hearing loss affecting higher frequencies first	g.6:113759212G > C	*Atp2b2*	*Atp2b2* < *Tkh* >	MGI:6470863	Missense	R969G	Semi- dominant	JM8A3.N1	No	Hypomorph
Twitch	*ttch*	*Arpc3*	MDLY	Complete deafness with vestibular dysfunction	g.16:18584128C > T	*Tbx1*	*Tbx1* < *ttch* >	MGI:6470864	Missense	D212N	Recessive	JM8A3.N1	No	Hypomorph
Jiggle	*jigl*	*Ccdc122*	MEWY	Complete deafness with vestibular dysfunction	Deletion between g.10:74,614,441 and g.10:74,635,149	*Pcdh15*	*Pcdh15* < *jigl* >	MGI:6470865	Deletion, with potential insertion	Loss of four exons towards 3' end of transcript	Recessive	JM8A1.N3	No	Similar
Rhythm	*rthm*	*Isg20*	MFFD	Complete deafness with vestibular dysfunction	g.18:57437258_57740507del	*Ctxn3, Ccdc192 and 6 noncoding genes*	*Del(18Ctxn3-Ccdc192)1Kcl*	MGI:6470868	Deletion	303 kb deletion	Recessive	JM8A3.N1	No	N/A
Rhyme	*rhme*	*Rhox13*	MEEK	Progressive hearing loss affecting higher frequencies first	g.10:20116294_20153024del	*Map3k5, Map7*	*Del(10Map3k5-Map7)2Kcl*	MGI:6470869	Deletion	36.7 kb deletion affecting last 9 exons of Map3k5 and first exon of Map7	Recessive	JM8A3.N1	No	Male sterility of *Map7* mutants is the same; hearing loss has not been reported
Spindizzy	*spdz*	*Slc35f2*	MHER	Complete deafness with vestibular dysfunction	Disruption between g.4:152,122,586 and g.4:152,123,017	*Espn*	*Espn* < *spdz* >	MGI:6470866	Deletion, with potential insertion or other disruption	Disruption in intron	Recessive	JM8A3.N1	No	Similar
Low frequency	*lowf*	*Mab21l4*	MCBX	Low frequency hearing loss	g.9:110455454C > A	*Klhl18*	*Klhl18* < *lowf* >	MGI:6470867	Missense	V55F	Recessive	JM8.F6	No	Similar
Low frequency	*lowf*	*Traf3ip3*	MATH	Low frequency hearing loss	g.9:110455454C > A	*Klhl18*	*Klhl18* < *lowf* >	MGI:6470867	Missense	V55F	Recessive	JM8.N4	No	Similar
Low frequency	*lowf*	*Mad2l2*	MBYL	Low frequency hearing loss	g.9:110455454C > A	*Klhl18*	*Klhl18* < *lowf* >	MGI:6470867	Missense	V55F	Recessive	JM8.F6	No	Similar
Low frequency	*lowf*	*Kazn*	MCFF	Low frequency hearing loss	g.9:110455454C > A	*Klhl18*	*Klhl18* < *lowf* >	MGI:6470867	Missense	V55F	Recessive	JM8.N4	No	Similar
Low frequency	*lowf*	*Sesn3*	MCKG	Low frequency hearing loss	g.9:110455454C > A	*Klhl18*	*Klhl18* < *lowf* >	MGI:6470867	Missense	V55F	Recessive	JM8.N4	No	Similar
Low frequency	*lowf*	*Pabpc1l*	MCRU	Low frequency hearing loss	g.9:110455454C > A	*Klhl18*	*Klhl18* < *lowf* >	MGI:6470867	Missense	V55F	Recessive	JM8A3.N1	No	Similar
Low frequency	*lowf*	*Kcne2*	MCSJ	Low frequency hearing loss	g.9:110455454C > A	*Klhl18*	*Klhl18* < *lowf* >	MGI:6470867	Missense	V55F	Recessive	JM8.N4	No	Similar
Low frequency	*lowf*	*Ptpn2*	MCTP	Low frequency hearing loss	g.9:110455454C > A	*Klhl18*	*Klhl18* < *lowf* >	MGI:6470867	Missense	V55F	Recessive	JM8A3.N1	No	Similar
Low frequency	*lowf*	*Tnfaip1*	MDEU	Low frequency hearing loss	g.9:110455454C > A	*Klhl18*	*Klhl18* < *lowf* >	MGI:6470867	Missense	V55F	Recessive	Unknown (imported line)	Unknown	-
Variable thresholds	*vthr*	*Dusp3*	MBVF	Variable moderate to severe hearing loss across all frequencies	Unknown	Unknown	-	-	-	-	-	JM8.N19	Unknown	-
-	Un- known	*Pdzd3*	METD	Low frequency hearing loss	Unknown	Unknown	-	-	-	-	-	JM8A1.N3	Unknown	-
-	Un- known	*Trim66*	MCVL	Low frequency hearing loss	Unknown	Unknown	-	-	-	-	-	JM8.N4	Unknown	-
-	Un- known	*Tpi1*	MCFC	High-frequency hearing loss	Unknown	Unknown	-	-	-	-	-	JM8.N4	Unknown	-
-	Un- known	*Aff3*	MCND	High-frequency hearing loss	Unknown	Unknown	-	-	-	-	-	JM8.N4	Unknown	-
-	Un- known	*Wdtc1*	MFYJ	High-frequency hearing loss, only seen in females	Unknown	Unknown	-	-	-	-	-	JM8.N4	Unknown	-
-	Un- known	*Ddah1*	MGKQ	High-frequency hearing loss	Unknown	Unknown	-	-	-	-	-	JM8A3.N1.C2	Unknown	-
-	Un- known	*Mir32*	MOAA	High-frequency hearing loss	Unknown	Unknown	-	-	-	-	-	JM8A3	Unknown	-
-	Un- known	*Sfxn3*	MUBE	Severe & profound hearing loss	Unknown	Unknown	-	-	-	-	-	JM8A3.N1	Unknown	-

This table summarises the 25 lines in which hearing phenotypes were observed which did not segregate with the targeted allele. While some of the targeted genes have been associated with a hearing phenotype (eg *Sesn3* homozygotes have abnormal waveforms [11], and *ISG20* has been associated with human hearing loss via genome-wide association studies (GWAS) [13, 14]), the targeted alleles did not segregate with the phenotypes reported in this paper and are not linked to the observed hearing loss described here

### ***The Klhl18***^***lowf***^*** allele (MCBX colony): low frequency progressive hearing loss***

**Fig. 2:**
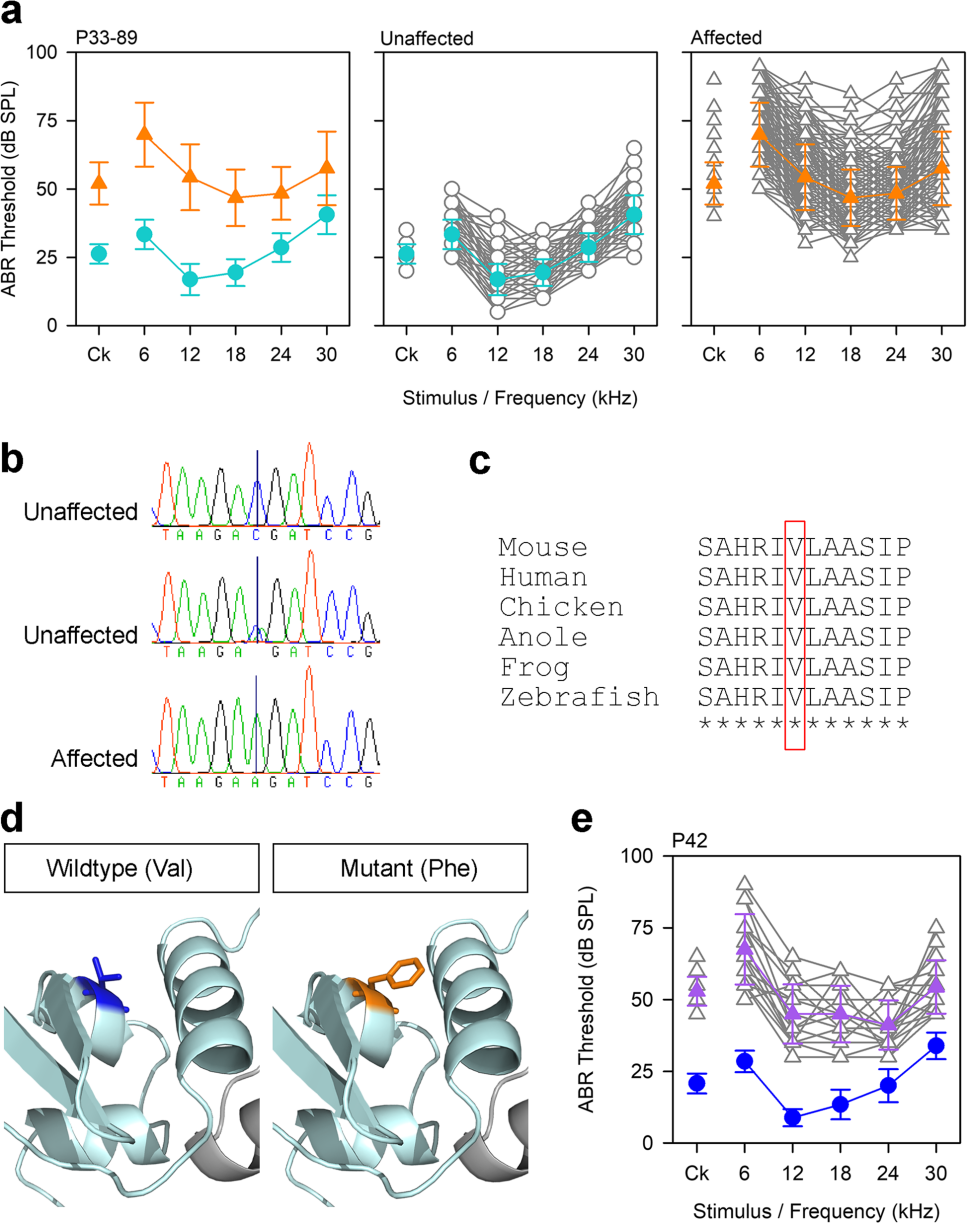
Mutations in *Klhl18* cause low frequency hearing loss. **a** Mean ABR thresholds from mice tested during establishment of the breeding colony derived from the MCBX colony. Mice were tested between 33 and 89 days old, and grouped into affected (*n* = 221, orange triangles) and unaffected (*n* = 213, teal circles) based on the bimodal distributions of thresholds for clicks and 6 kHz stimuli. Plots of individual ABR thresholds (grey) are shown separately with the mean trace indicated by coloured lines and symbols; error bars on mean trace are standard deviations. **b** Sequence traces from two unaffected mice (one wildtype, one heterozygote) and an affected mouse (homozygote) showing the variant *Klhl18*^*lowf*^ (MCBX colony), p.V55F. **c** Clustal alignment from mouse, human, chicken, anole lizard, frog and zebrafish showing that the affected amino acid is highly conserved (red box). **d** Close-up of the BTB domain (pale cyan) showing the amino acid structures for the wildtype residue (blue, left) and the mutant residue (orange, right). **e** Mean ABR thresholds from mice heterozygous for the *lowf* allele (*n* = 13, blue circles) and compound heterozygotes carrying the *lowf* allele and the *Klhl18*^*tm1a*^ allele (*n* = 17, purple triangles), demonstrating the low frequency hearing loss phenotype. Individual traces for the compound heterozygotes are shown in grey; error bars on mean trace are standard deviations. Data underlying plots in this figure are in Additional File 3

Mice homozygous for this mutation displayed the unusual phenotype of low frequency hearing loss (*lowf*) (Fig. 2a). We mapped the mutation to a 5.7 Mb region on chromosome 9 (Additional File 1: Fig. S1), in which we found 3 exonic variants (Additional File 2: Table S1), two of which proved to be false calls when resequenced using Sanger sequencing. The third variant was a missense variant in the gene *Klhl18*, g.9:110455454C > A, causing an amino acid change of p.(Val55Phe) (ENSMUST00000068025) (Fig. 2b, c). We used Phyre2 [15] to create a model of KLHL18 based on two structures, a *Plasmodium falciparum* Kelch protein [16] and the crystal structure of the BTB-BACK domains of human KLHL11 [17] (Fig. 2d). The affected residue lies in the BTB domain, which is involved in protein–protein interaction, including homodimerization [18–20]. The mutant phenylalanine, being much larger than the wildtype valine, could potentially disrupt the BTB domain and thus protein function. We confirmed that this was the causative mutation by complementation testing with mice carrying the *Klhl18*^*tm1a(KOMP)Wtsi*^ targeted allele [9, 11] (Fig. 2e). Middle ear dissection and inner ear clearing showed no gross malformations of the ossicles or the inner ear (Additional File 1: Figs S2, S3). This phenotype was observed in eleven different lines in total (Fig. 1), so we sequenced affected mice from eight more of these lines (in addition to the MCBX line) and discovered that all the affected mice (*n* = 15) were homozygous for the *Klhl18*^*lowf*^ allele, while unaffected littermates (*n* = 8) were heterozygous or wildtype. We were not able to obtain DNA samples from affected mice of the remaining two lines (MCVL, METD). Further characterisation of the unusual *Klhl18*^*lowf*^ phenotype is described in [21].

### ***The Atp2b2***^***Tkh***^*** allele (MEBJ colony): semidominant progressive hearing loss***

**Fig. 3:**
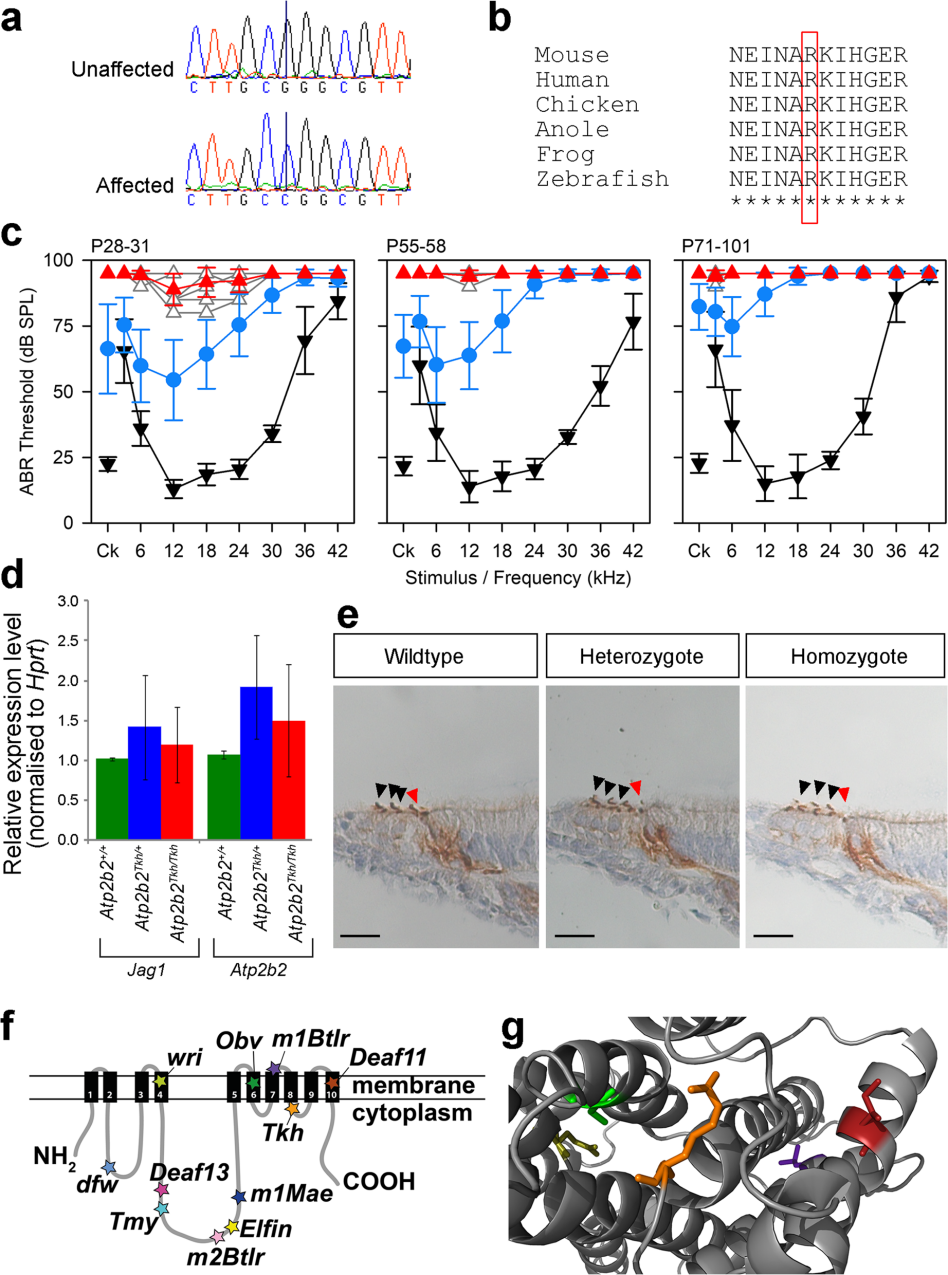
A missense mutation in Atp2b2 results in semidominant progressive hearing loss. **a** Sequence traces from an unaffected and an affected mouse showing the variant *Atp2b2*^*Tkh*^ (MEBJ colony), p.R969G. **b** Clustal alignment from mouse, human, chicken, anole lizard, frog and zebrafish showing that the affected amino acid is highly conserved (red box). **c** Mean ABR thresholds from wildtype (black inverted triangles), heterozygote (blue circles) and homozygote (red triangles) mice at P28-P31 (*n* = 10 wildtypes, 27 heterozygotes, 9 homozygotes), P55-P58 (*n* = 9 wildtypes, 24 heterozygotes, 6 homozygotes) and P71-P101 (*n* = 9 wildtypes, 24 heterozygotes, 6 homozygotes). Error bars are standard deviations. **d** *Jag1* and *Atp2b2* qPCR on RNA from the organ of Corti at P4 (*n* = 6 wildtypes, 6 heterozygotes and 6 homozygotes). There was no difference between the *Jag1* levels of wildtypes, heterozygotes and homozygotes (*p* = 0.307, Welch’s one-way ANOVA). We found a marginally significant difference in *Atp2b2* levels (*p* = 0.036, Welch’s one-way ANOVA), but this was not borne out by the post hoc Games-Howell multiple comparison test (*p* = 0.052 for wildtypes compared to heterozygotes, *p* = 0.379 for wildtypes compared to homozygotes, *p* = 0.553 for heterozygotes compared to homozygotes). The bars show the mean expression levels, and error bars are standard deviations. **e** PMCA2 antibody stains at P4 (*n* = 3 wildtypes, 3 heterozygotes, 3 homozygotes), showing hair cells from the region 43% of the distance along the organ of Corti from base to apex. Images are representative examples for each genotype, and no differences were observed between genotypes. Arrowheads indicate the hair cells, red for the inner hair cell and black for the outer hair cells. Scale bar = 20 µm. **f** Schematic of PMCA2 protein showing the 10 transmembrane helices and the 11 known missense mutations [24, 25, 29–34, 36]). **g** Model of PMCA2 protein with the amino acid affected by the *Tkh* allele in orange. The four other missense mutations located in or near the transmembrane helices are also visible, shown in green (*Obv *[31]), dark red (*Deaf11* [25]), olive yellow (*wri* [33]) and purple (*M1Btlr *[29]). The wildtype amino acid structures are shown for all four residues. Data underlying plots in this figure are in Additional File 3

The Tikho (*Tkh*, Russian for “quiet”) mutation was mapped to a 5.2 Mb region on chromosome 6 (Additional File 1: Fig. S1). We found one exonic single-nucleotide variant (SNV) in this region (Additional File 2: Table S1), a missense mutation in the *Atp2b2* gene, g.6:113759212G > C, causing an amino acid change of p.(Arg969Gly) (ENSMUST00000101045) (Fig. 3a, b). Mice homozygous for this allele exhibited rapidly progressive hearing loss, while heterozygotes displayed slower progressive hearing loss, with the high frequencies affected first (Fig. 3c, Additional File 1: Fig. S4). Homozygotes and heterozygotes displayed normal gait and balance. We sequenced mice from the breeding colony and confirmed the segregation of the allele with the phenotypes observed. No gross malformations of the ossicles or inner ear were observed (Additional File 1: Figs S2, S3). We used quantitative PCR (qpCR) to test RNA expression and immunohistochemistry to study protein localisation, but found no difference between wildtypes, heterozygotes and homozygotes in either test (Fig. 3d, e), suggesting that the variant does not affect mRNA transcription or protein localisation. We modelled the mutation using a model of the rabbit skeletal muscle Ca^2+^-ATPase [22], which matches 75% of the residues with 100% confidence. This includes the mutant residue, which lies in the cytoplasmic domain between transmembrane domains 8 and 9. There are 14 reported *Atp2b2* mouse mutants with a hearing phenotype, 10 of which result from a single amino acid change (Fig. 3f [23]). Only the *Deaf11* and *Deaf13* missense alleles are like the *Tkh* allele in that they do not result in ataxia in homozygotes [24–36]. The three closest missense mutations are *Obv*, *m1Btlr* and *Deaf11*, all of which lie within 100 amino acids of the mutated *Tkh* residue (Fig. 3f, g).

### The Del(10Map3k5-Map7)2Kcl allele (MEEK colony): progressive hearing loss and male sterility

**Fig. 4:**
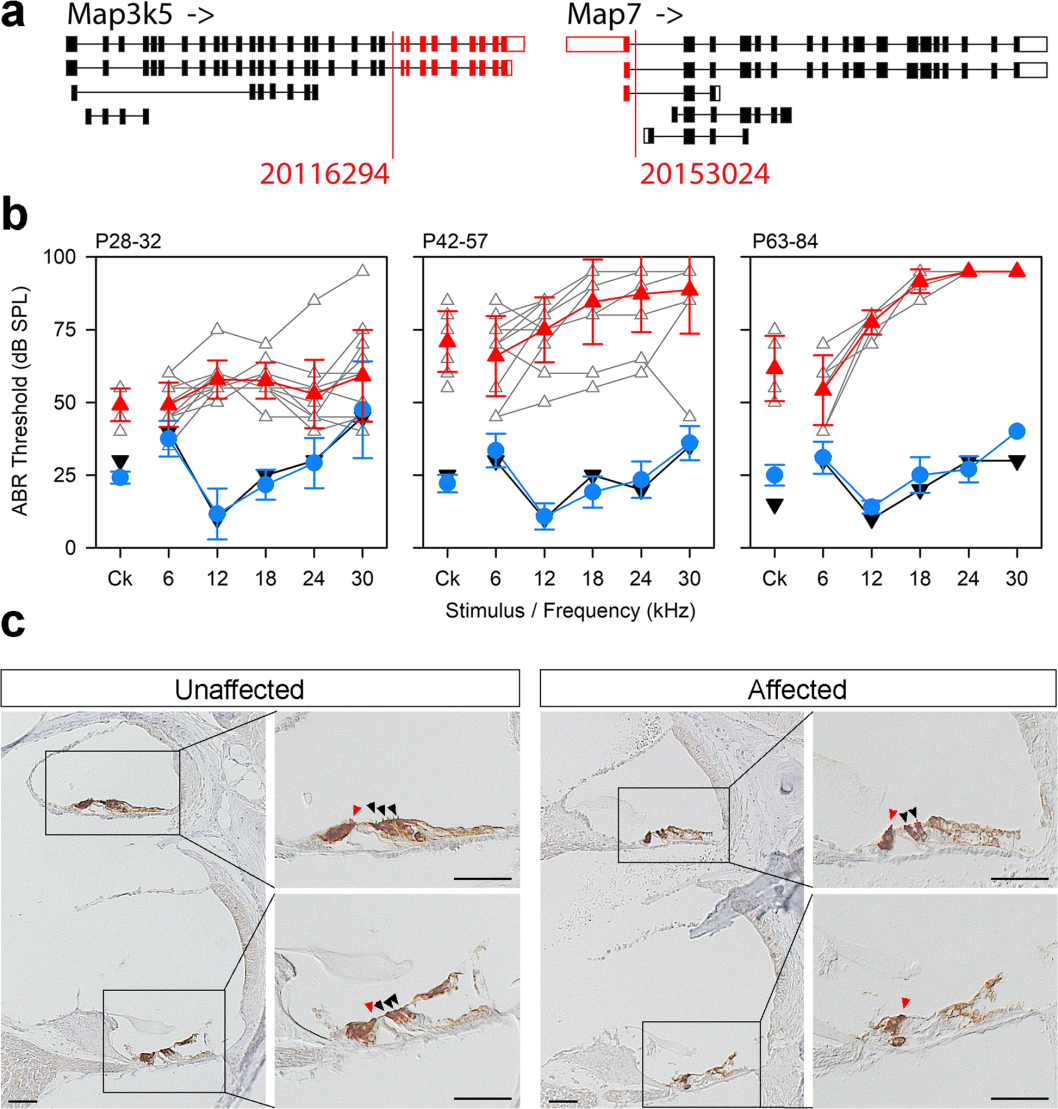
The *rhme* deletion spanning *Map3k5* and *Map7* causes progressive hearing loss and disrupts the organ of Corti. **a** Schematic of the *rhme* deletion seen in the MEEK line showing the different isoforms of the two affected genes (not to scale). Red indicates missing exons. **b** Mean ABR thresholds showing progressive hearing loss in mice homozygous for the deletion (wildtype shown by black inverted triangles, heterozygote by blue circles, homozygotes by red triangles) (*n* = 1 wildtype, 6 heterozygotes and 12 homozygotes at P28-32; 1 wildtype, 19 heterozygotes and 11 homozygotes at P42-57; 1 wildtype, 5 heterozygotes and 6 homozygotes at P63-84 (see Additional File 1: Fig. S9a for individual thresholds)). Traces from individual homozygotes are shown in grey; error bars on mean trace are standard deviations. c Expression of MYO7A, a hair cell marker, in the organ of Corti of adult mice showing an apical turn (top; 94% of the distance along the organ of Corti from base to apex), where the hair cells are still present in affected mice, and a mid-basal turn (bottom, 43% of the distance from base to apex), where only the inner hair cell is identifiable (hair cells are marked with arrowheads; red for inner hair cells, black for outer hair cells). Brown indicates where MYO7A is expressed. Three affected and 3 unaffected littermates (at matched ages between 33 and 84 days old) were examined, and the images shown are representative of our observations. Scale bar = 50 µm. Data underlying plots in this figure are in Additional File 3

The rhyme (*rhme*) allele was observed to cause male sterility; no pregnancies or pups were obtained from twelve different affected males, paired in matings for at least 66 days. Affected females were fully fertile. We mapped the mutation to a 7 Mb region on chromosome 10 (Additional File 1: Fig. S1), but there were no exonic variants in the region, only an interchromosomal translocation from chr10 to chr13 predicted by BreakDancer (Additional File 2: Table S1), which proved to be a false call when we carried out Sanger sequencing across the predicted breakpoint. We then looked for deletions of whole exons, using the Integrative Genomics Viewer (IGV) to view all reads aligned to the region, and found a large candidate deletion which we confirmed by segregation testing in phenotyped mice. The causative mutation is a 36.7 kb deletion, g.10:20116294_20153024del, which includes the last 9 exons of *Map3k5* and the first exon of *Map7* (Fig. 4a). Mutations in *Map7* have been associated with male sterility [37, 38], but *Map3k5* homozygote mice have been reported to be fertile [39], suggesting that the loss of the first exon of *Map7* is affecting MAP7 protein function, resulting in the observed infertility in *rhme* affected males. Male and female homozygotes for the *rhme* deletion had raised thresholds at high frequencies at 4 weeks old, and the hearing loss progressed with age (Fig. 4b, Additional File 1: Fig. S4). No gross malformations of the ossicles or inner ear were observed (Additional File 1: Figs S2, S3). We investigated the hair cells of affected adults using immunohistochemistry and found the organ of Corti was disrupted towards the basal regions, with loss of outer hair cells and disruption and collapse of the tunnel of Corti (Fig. 4c). We investigated the expression of *Map7* and *Map3k5* using single-cell RNA sequencing (RNAseq) data from the gEAR database [40] and found that while *Map3k5* showed low expression in most cell types of the cochlear duct, *Map7* was expressed in multiple cell types, in particular the outer hair cells and outer pillar cells, increasing over time in both. *Map7* is also expressed in the lateral wall, although not as strongly (Additional File 1: Fig. S5).

### ***The Tbx1***^***ttch***^*** allele (MDLY colony): complete deafness with vestibular dysfunction***

**Fig. 5:**
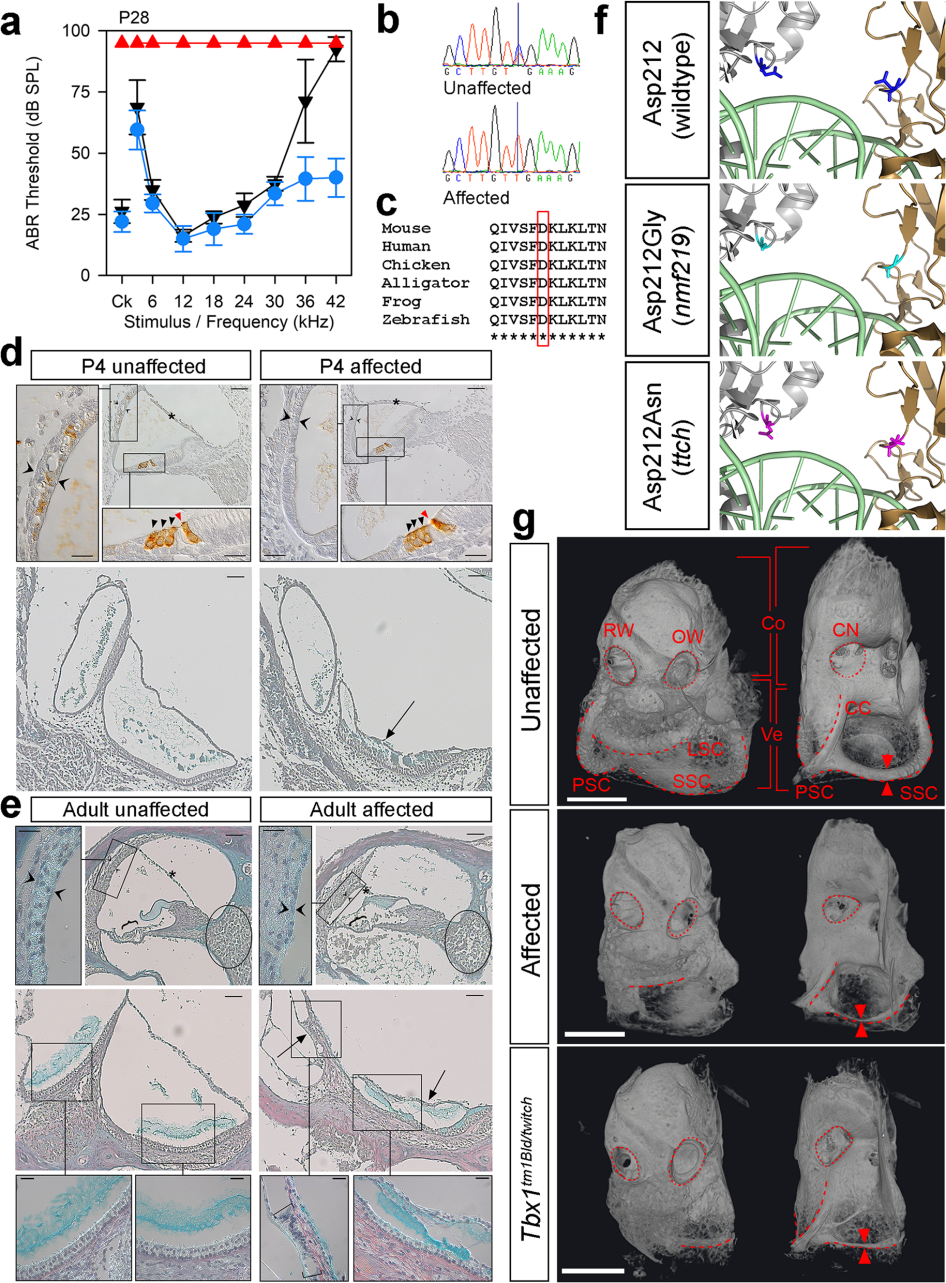
A missense mutation in Tbx1 causes malformation of the semicircular canals and profound deafness. **a** Mean ABR thresholds from P28 mice homozygous (*n* = 6, red triangles), heterozygous (*n* = 10, blue circles) and wildtype (*n* = 4, black inverted triangles) for the *Tbx1*^*ttch*^ allele (MDLY colony), p.D212N. Error bars are standard deviations. See Additional File 1: Fig. S9b for individual thresholds. **b** Sequence traces from an unaffected and an affected mouse showing the variant. **c** Clustal alignment showing conservation of the affected amino acid (red box). **d** P4 sections showing the cochlear duct (top, anti-MYO7A brown stain, blue counterstain, 43% of the distance along the organ of Corti from base to apex) and the maculae (bottom, trichrome staining) (*n* = 3 affected, 3 unaffected littermates). MYO7A is expressed in hair cells (arrowheads; red/black for inner/outer hair cells) and the intermediate cells of the stria vascularis. **e** Trichrome-stained sections from adult mice (*n* = 4 affected, 4 unaffected littermates) showing the cochlear duct (top, 72% of the distance along the organ of Corti from base to apex) and the maculae (bottom). Brackets mark the organ of Corti and the spiral ganglion area is circled. Square brackets indicate abnormal saccular hair cells. In **d** and **e**, main panel scale bars = 50 µm, high magnification panel scale bars = 20 µm. Asterisks mark Reissner’s membrane, twin open arrowheads the stria vascularis, and arrows the collapsed saccule. **f** Human TBX1 protein model [104], as a homodimer (silver, gold) bound to DNA (pale green). The Asp212 residue is marked in blue (top), with the *nmf219* mutant residue [41] in cyan (middle) and the *ttch* mutant residue in magenta (bottom). **g** MicroCT scans of cleared inner ears from affected P28 mice (*n* = 3, middle) and compound heterozygotes (*Tbx1*^*tm1Bld/ttch*^) at P21 (*n* = 2, bottom). An unaffected P21 mouse is shown at the top (P21 *n* = 4; P28 *n* = 3). Dashed lines outline the semicircular canals, with twin arrowheads for comparison of their width. The middle ear side, with the round (RW) and oval (OW) windows (dotted lines) is on the left, and the brain side, where the cochlear nerve exits (CN, dotted lines), on the right. Brackets indicate the cochlea (Co) and vestibular region (Ve). LSC = lateral semicircular canal; SSC = superior semicircular canal; PSC = posterior semicircular canal, CC = common crus. Scale bar = 1 mm. Data underlying plots in this figure are in Additional File 3

Mice homozygous for the twitch (*ttch*) allele exhibited circling and head bobbing behaviour and had no response to any stimulus up to 95 dB, the maximum sound output of our equipment (Fig. 5a). The ossicles were normal in appearance (Additional File 1: Fig. S2), but we observed signs of inflammation in the middle ear at postnatal day (P)28 (serous effusion, thickened epithelia and capillary hyperplasia) which were more common in affected mice. The gross morphology of the vestibular region was severely affected, with very thin or absent semicircular canals (Fig. 5g). The inner ears of P4 pups displayed a reduced scala media and a thinner stria vascularis at early postnatal stages, although the hair cells appear to have developed normally (Fig. 5d). The saccule had collapsed, but the utricular lumen remained open (Fig. 5d). At adult stages, the scala media was even smaller, with a thin or absent stria vascularis, a collapsed Reissner’s membrane, extensive degeneration of the organ of Corti, and spiral ganglion cell loss (Fig. 5e). Both utriculus and sacculus had collapsed, and the hair cells in the utricle appeared disorganised (Fig. 5e). We mapped the mutation to a 3 Mb region on chromosome 16 (Additional File 1: Fig. S1), which contained only 1 exonic SNV (Additional File 2: Table S1), which segregated with the phenotype in the colony. This was a missense variant in *Tbx1*, g.16:18584128C > T, which results in an amino acid change of p.(Asp212Asn) (ENSMUST00000232335) (Fig. 5b, c). Asp212 is the same amino acid affected by the recently-reported nmf219 mutation, which has a very similar inner ear phenotype [41], although the underlying coding change (g.16:18584127 T > C), and the resulting amino acid change (Asp212Gly), both differ (Fig. 5f). TBX1 binds to DNA as a dimer, and Asp212 is located between the two monomers, so amino acid changes could potentially affect the structure of the dimer and its capacity to bind DNA [41] (Fig. 5c). Many other *Tbx1* mutant alleles are homozygous perinatal lethal [42–45], with gross morphological abnormalities evident as early as embryonic day (E) 8.5 [43], but neither of these Asp212 mutants show reduced viability of homozygotes. We carried out a complementation test with the *Tbx1*^*tm1Bld*^ allele [45] and found that the inner ears of compound heterozygotes were notably smaller than those of littermates carrying one copy of the *ttch* allele and had malformed semicircular canals (Fig. 5f). This phenotype, which resembled that of *Tbx1*^*ttch*^ homozygotes, confirmed that the *ttch* allele is the causative mutation.

We also identified two other variants in this line before the non-recombinant region was fully defined; a 27 bp inframe deletion in the *Kmt2d* gene which underlies Kabuki syndrome in humans [46] (Additional File 1: Fig. S6a), and a missense mutation in the gene *Muc13* (Additional File 1: Fig. S6c, d). These mutations were confirmed to be present in multiple mice from the MDLY colony but did not segregate with the *ttch* phenotype, and follow-up ABR tests found no effect on hearing in homozygotes of either mutation (Additional File 1: Fig. S6b, e, g). We investigated the expression of MUC13 in the cochlea at P4 and found it was expressed in hair cells, pillar cells and the basal cells of the stria vascularis (Additional File 1: Fig. S6f).

### ***The Pcdh15***^***jigl***^*** allele (MEWY colony): complete deafness with vestibular dysfunction***

**Fig. 6:**
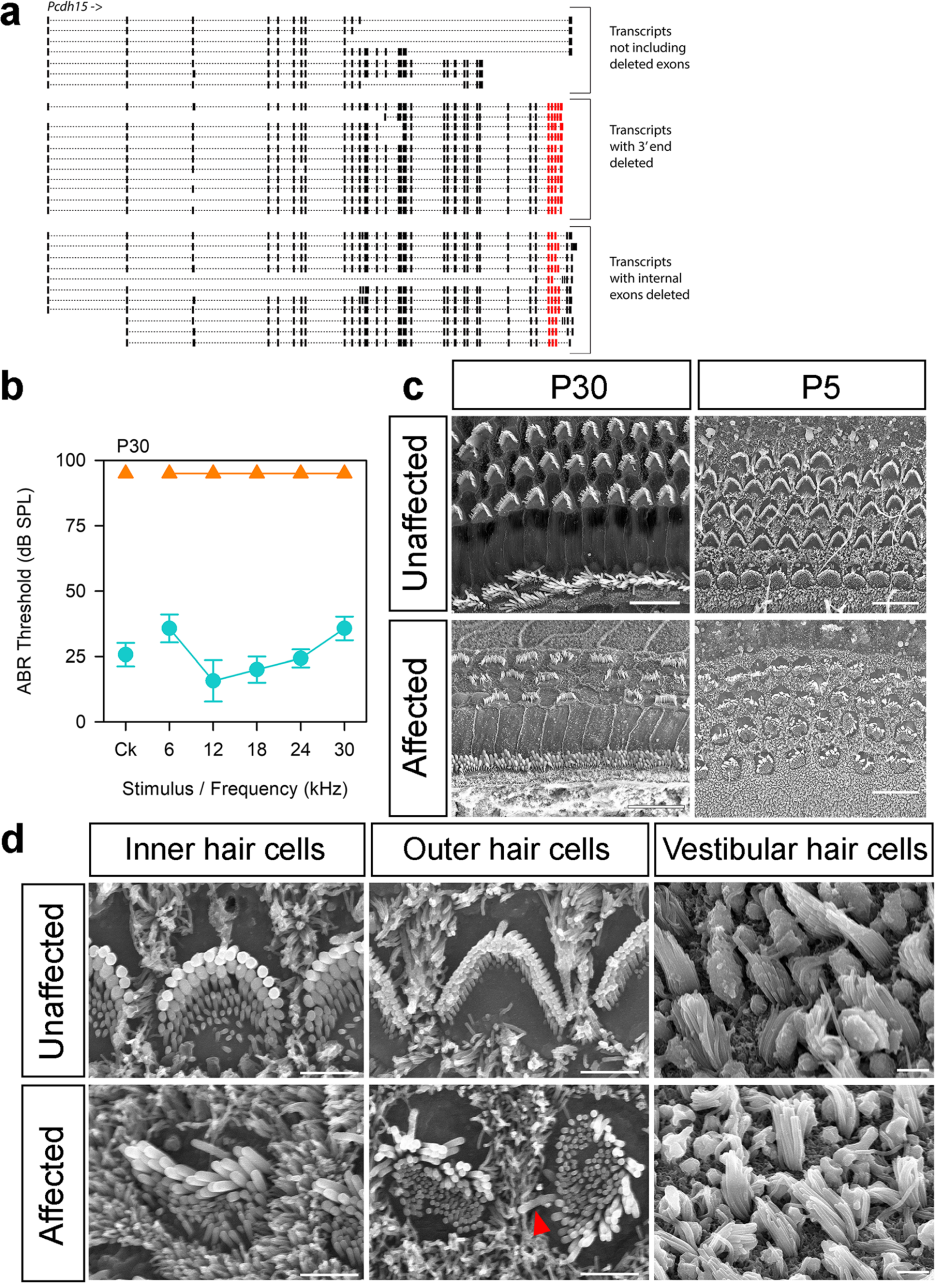
A deletion within the *Pcdh15* gene causes disruption of stereocilia bundles and profound deafness. **a** Schematic of *Pcdh15* isoforms showing the internal deletion in *Pcdh15* (*jigl*, MEWY colony*)*, showing the effects of the deletion on different transcripts (not to scale). Red indicates the deleted exons. Transcripts are shown in their entirety, but not all exons are visible due to scale. **b** Mean ABR thresholds of affected (*n* = 6, orange triangles) and unaffected (*n* = 7, teal circles) mice at P30. Error bars are standard deviations. **c** Scanning electron micrographs of the organ of Corti at P30 (60–70% of the distance from base to apex, scale bar = 10 µm, *n* = 3 unaffected, 6 affected mice) and P5 (50–80% of the distance from base to apex, scale bar = 10 µm, *n* = 5 unaffected, 4 affected mice). Mice at P5 were not able to be phenotyped by circling behaviour, but the scanning electron micrographs showed a clear bimodal distribution of affected and unaffected based on disorganisation of hair bundles, and we were unable to amplify the deleted region in affected pups (see Table S7 for primers). Representative examples are shown here. Affected P5 mice had less well-organised hair cell bundles than unaffected littermates. The overall “V” shape of the stereocilia bundle was distorted and irregular in the organ of Corti of affected mice. The polarity of some outer hair cell bundles was rotated by up to 90°, and in some cases, the kinocilium was on the opposite side of the cell (arrowhead in **d**). **d** Close ups of hair cells at P5, showing cochlear hair cells from the same region as in **c**, and vestibular hair cells from the macula (*n* = 2 unaffected and 2 affected mice). The arrowhead indicates an example of a kinocilium on the opposite side of the outer hair cell from the stereocilia bundle. Macular stereocilia bundles seemed to have a more ordered staircase structure in the mice classed as unaffected compared to affected mice, where all stereocilia appeared to be long with little sign of a staircase arrangement. Scale bars = 2 µm. Data underlying plots in this figure are in Additional File 3

The jiggle (*jigl)* allele was mapped to a 26.9 Mb region on chromosome 10 (Additional File 1: Fig. S1) in which we found no small exonic variants, but one potential interchromosomal translocation within the gene *Eef2* was identified by BreakDancer (Additional File 2: Table S1). We investigated this using IGV and found that it was based on a small percentage of the reads covering the region, most of which were correctly mapped, suggesting it was a false call. We then examined the entire non-recombinant region and identified a deletion towards the 3’ end of *Pcdh15*, which includes up to 6 coding exons depending on the transcript. There are 29 protein-coding isoforms of *Pcdh15* (ensembl.org, accessed July 2021), 22 of which contain the affected exons. The deletion results in the loss of the 3’ end of the coding sequence for eleven of those transcripts, and in the loss of internal exons for the remaining eleven (Fig. 6a). We localised the 5’ breakpoint to the region between 10:74,614,441 and 10:74,619,826, and the 3’ breakpoint to the region between 10:74,634,994 and 10:74,635,149. We used primers designed to amplify a region within the deletion (Additional File 2: Table S3) to confirm it was not present in 20 affected animals, with 20 unaffected mice from the MEWY colony as controls. *Pcdh15*^*jigl*^ homozygotes display circling and head bobbing and have no auditory brainstem response to any stimulus up to 95 dB (Fig. 6b). There were no gross malformations of the ossicles or inner ear (Additional File 1: Figs S2, S3). Scanning electron microscopy of the organ of Corti at P30 showed that affected mice exhibited hair bundle disorganisation that was most marked in the outer hair cells (Fig. 6c). We observed a similar phenotype in the P5 organ of Corti, with disorganisation of the stereocilia within hair cell bundles, a distorted bundle shape overall, and disoriented bundles (Fig. 6c, d). Hair bundles in the P5 vestibular maculae also lacked the typical staircase organisation (Fig. 6d). These hair bundle defects are similar to those described in other *Pcdh15* mutants, which also exhibit deafness [47, 48] and reflect the role of PCDH15 as a component of the tip links between adjacent stereocilia [49, 50].

### The Del(18Ctxn3-Ccdc192)1Kcl allele (MFFD colony): complete deafness with vestibular dysfunction

**Fig. 7:**
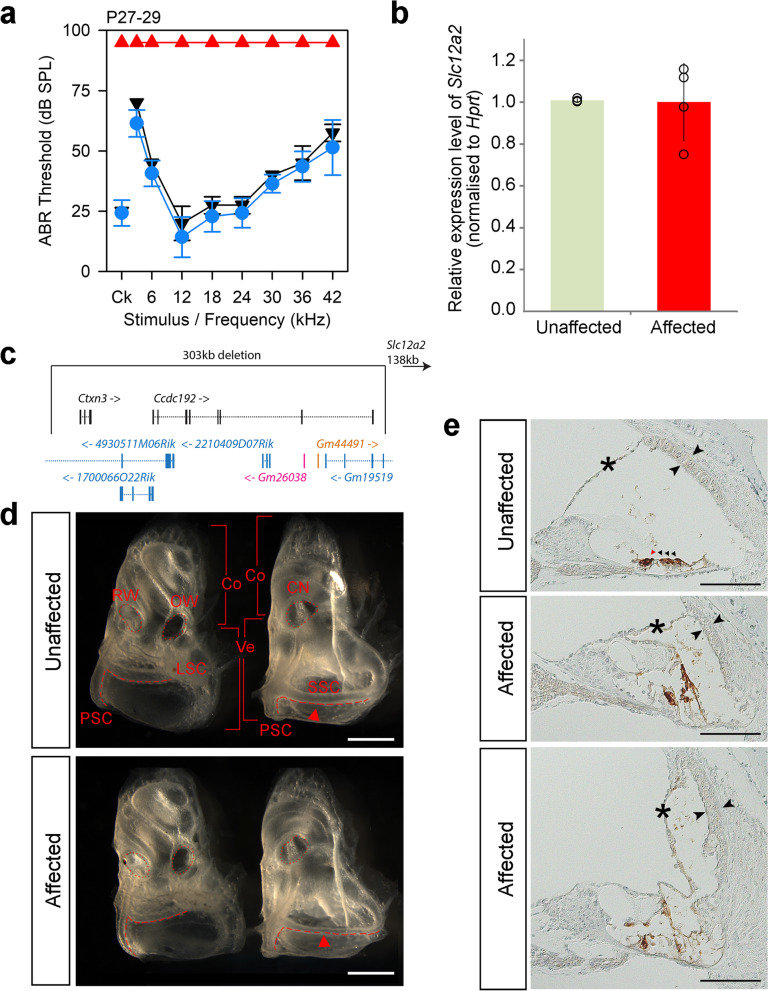
A 303 kb deletion on chr18 affects semicircular canals and results in profound deafness. **a** Mean ABR thresholds of mice homozygous (*n* = 6, red triangles), heterozygous (*n* = 7, blue circles) and wildtype (*n* = 2, black inverted triangles) for the *rthm* allele (MFFD colony) at P28 ± 1 day. Error bars are standard deviations. See Additional File 1: Fig. S9c for individual thresholds. **b** *Slc12a2* qPCR on RNA from the brains of P28 affected (*n* = 4, red, right) and unaffected (*n* = 4, green, left) mice. There is no significant difference between wildtypes and homozygotes (*p* = 1, Wilcoxon rank sum test). The bars show the mean expression level, and the points show the individual measures. Error bars are standard deviations. **c** Schematic showing the genes affected by the deletion. There are two protein-coding genes (black), one miRNA gene (orange), one snRNA gene (pink) and four lncRNA genes (blue). *Slc12a2* is located 138 kb downstream of the 3’ end of the deletion (indicated by arrow top right). **d** Inner ears from affected (*n* = 3) and unaffected (*n* = 3) mice at P28 ± 1 day. The middle ear side, with the round (RW) and oval (OW) windows is shown on the left of each panel, and the brain side, where the cochlear nerve exits (CN), on the right. The round and oval windows and the cochlear nerve exits are marked by dotted lines, and the semicircular canals by dashed lines. Brackets indicate the cochlea (Co) and vestibular region (Ve). LSC = lateral semicircular canal; SSC = superior semicircular canal; PSC = posterior semicircular canal. Scale bar = 1 mm.The superior semicircular canals are thinner in affected mice (red arrowheads). **e** Immunohistochemistry of the cochlear duct of affected (*n* = 3) and unaffected (*n* = 3) mice at P28. Brown indicates the presence of MYO7A, which marks hair cells and the intermediate cells of the stria vascularis (marked by twin open arrowheads). Hair cells are only clearly visible in the unaffected mouse, indicated by the arrowheads (red for the inner hair cell and black for the outer hair cells). Asterisks show the Reissner’s membrane, which is displaced in affected mice. The top two panels show the apical region, 72% of the distance from the base to the apex. The lower panel shows the basal region, at 16% of the base-apex distance. Scale bar = 100 mm. Data underlying plots in this figure are in Additional File 3

Mice homozygous for the rhythm (*rthm*) allele exhibited complete deafness (Fig. 7a) with circling and head bobbing, suggesting vestibular dysfunction. We mapped the mutation to a 3.3 Mb region on chromosome 18 (Additional File 1: Fig. S1) but did not identify any exonic variants in the region (Additional File 2: Table S1). We used IGV to examine the nonrecombinant region and discovered a 303 kb deletion on chromosome 18, g.18:57437258_57740507del, covering eight genes, including two protein-coding genes (*Ctxn3*, *Ccdc192*), four lncRNA genes, one miRNA and one snRNA (Fig. 7c). This deletion segregated with the phenotype within the colony. The ossicles appeared normal (Additional File 1: Fig. S2) but the lateral semicircular canal was thinner in homozygotes than in heterozygotes (Fig. 7d), and MYO7A staining revealed severe disruption of the cochlear duct (Fig. 7e). This phenotype is similar to that of mice mutant for *Slc12a2 *[51], which lies 138kbp 3’ of the deletion, so we extracted RNA from brain tissue from affected mice and their unaffected littermates at 4 weeks old, and carried out qPCR to determine whether *Slc12a2* was misregulated. However, there were no significant differences in the levels of *Slc12a2* expression in homozygotes compared to heterozygote littermates (Fig. 7b). Thus, we found no evidence of a position effect of the deletion on *Slc12a2* expression in brain. It is possible that the deletion only affects *Slc12a2* expression in the ear, but the stria vascularis is still present in affected *rthm* mice (Fig. 7e) and it is highly abnormal in *Slc12a2* mutants [51], supporting the hypothesis that *Slc12a2* is not involved in this phenotype despite its proximity to the *rthm* deletion. We investigated the expression of the eight genes in the deletion using the gEAR database [40], but found data for only one, *Ctxn3*, which is strongly expressed in the basal cells of the stria vascularis and in fibrocytes of the lateral wall of the cochlea at P30 and in type I fibrocytes at P20 (Additional File 1: Fig. S5).

### ***The Espn***^***spdz***^*** allele (MHER colony): complete deafness with vestibular dysfunction***

**Fig. 8:**
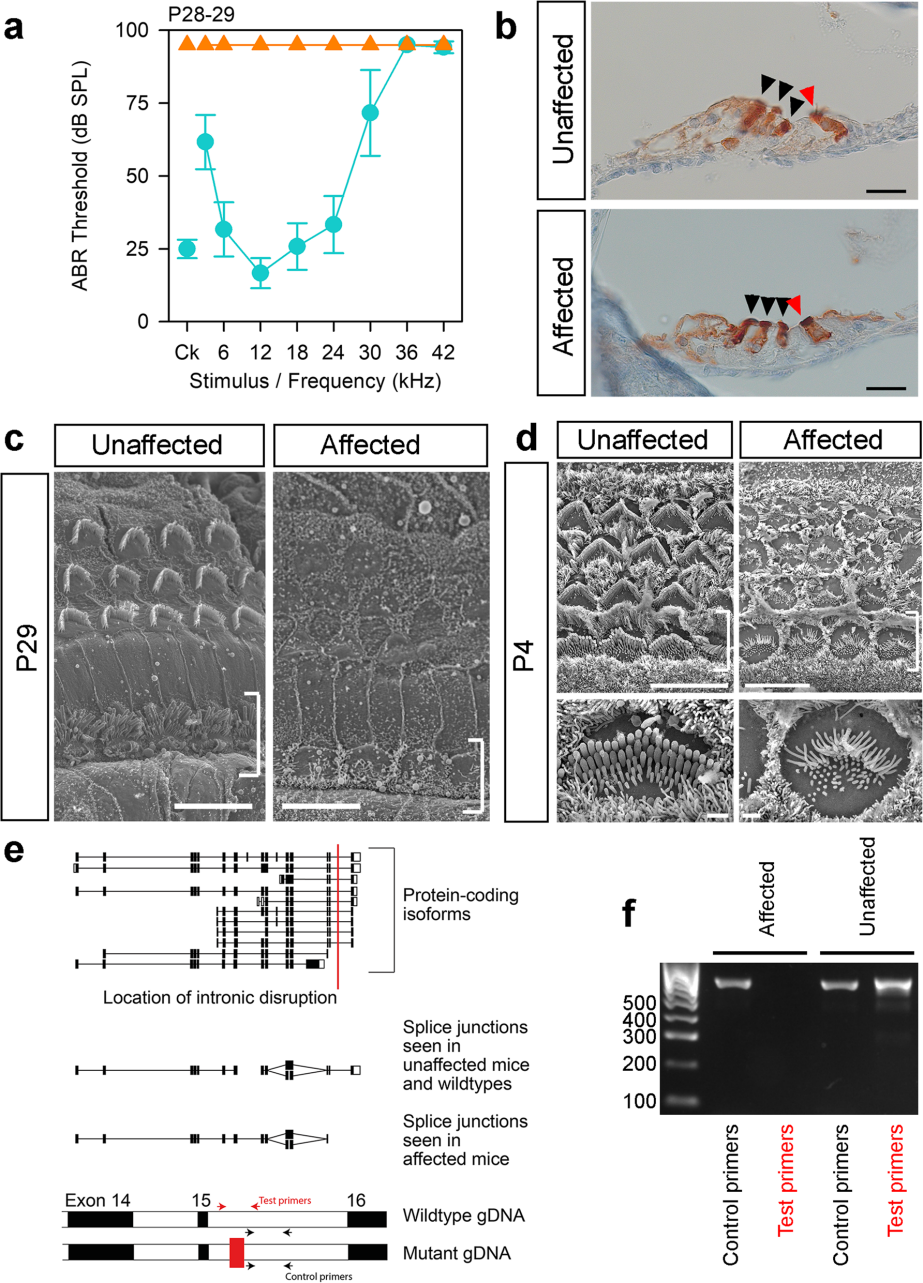
Mice carrying the *spdz* allele (MHER colony) are profoundly deaf, with abnormal hair cell stereocilia at P5 and hair cell degeneration by P29. **a** Mean ABR thresholds of affected (orange triangles, *n* = 7) and unaffected (teal circles, *n* = 6) mice at P28-29. Error bars are standard deviations. **b** Expression of MYO7A (brown) in hair cells from the apical region of the organ of Corti (90–95% of the distance from base to apex) of mice at P28 (*n* = 3 affected, 3 unaffected), showing that hair cells are visible in affected mice (arrowheads, red for the inner hair cell and black for outer hair cells). Scale bar = 20 µm. **c** Scanning electron micrographs of the 12 kHz best-frequency region (65–70% of the distance from base to apex) of the organ of Corti in unaffected (*n* = 4) and affected (*n* = 3) mice at P29. Brackets indicate the inner hair cell row. Most hair bundles are missing. Scale bar = 10 µm. **d** Scanning electron micrographs of a homozygous mutant mouse at P4 (*n* = 3) and an age-matched wildtype, showing the 42 kHz best-frequency region (20% of the distance from base to apex). Brackets indicate the inner hair cell row; scale bar = 10 µm. Lower panels show a magnified view of inner hair cells in the same region (scale bar = 1 µm). **e** Schematic showing the known protein-coding isoforms of *Espn* and the location of the intronic disruption (red line). The splice junctions identified in brain cDNA are shown below; each line indicates a splice junction visible in at least one mouse. At the bottom is a schematic of the genomic DNA (gDNA) showing exons 14, 15 and the start of exon 16 in black, separated by introns in white (not to scale). The disruption in the mutant is shown by the addition of a red block in the intron. The “test” primer pair (red arrows) fails to amplify in mutant gDNA, and the “control” primer pair (black arrows) works for both wildtype and mutant. **f** Gel showing bands amplified using these primer pairs (Additional File 2: Table S3) on an affected mouse and an unaffected mouse. The size of ladder bands is shown at the side in bp. Data underlying plots in this figure are in Additional File 3

Mice homozygous for this mutation displayed circling and head bobbing and had no response to sound up to 95 dB (Fig. 8a); the allele was named spindizzy (*spdz*). There were no gross malformations of the ossicles and inner ear (Additional File 1: Figs S2, S3), and MYO7A staining in adults suggested hair cells were present (Fig. 8b). However, scanning electron microscopy showed that there were no stereocilia bundles visible at P28 in homozygotes (Fig. 8c), and at P4, stereocilia bundles were present but disorganised, with thin stereocilia and ectopic stereocilia rows (Fig. 8d). The mutation mapped to a 3 Mb region on chromosome 4 (Additional File 1: Fig. S1) containing 28 protein-coding genes. There were 13 small variants called by Samtools in this region (Additional File 2: Table S1), none of which were within coding sequence. BreakDancer detected four intrachromosomal rearrangements (Additional File 2: Table S1), affecting exons of *Klhl21*, *Nol9*, and *Acot7*, and an intron of *Plekhg5*, but all four are based on a very low proportion of the reads in each homozygote. The only known deafness gene in the non-recombinant region is *Espn*, mutations in which result in shorter, thinner stereocilia at birth, followed by degeneration of stereocilia in early adulthood [52–54]. We therefore sequenced *Espn* mRNA from the brains of adult affected mice and their unaffected littermates. No splicing errors were observed for most of the exons; however, we were unable to amplify sequence from exons 15 and 16 in homozygotes (Fig. 8e). Exon 15 (ENSMUSE00001290053) is a 12 bp exon located 85 bp from exon 14 and separated by 2.35 kb from exon 16, which is the final exon (Fig. 8e). We resequenced the genomic region between exons 14 and 16 in three *spdz* homozygotes using Sanger sequencing, and while no variants were observed in intron 14–15 or in exon 15, we were unable to amplify a 431 bp region in the intron between exons 15 and 16, between g.4:152,122,586 and g.4:152,123,017, in any of the homozygotes. We successfully sequenced this region in two wildtype mice. This failure to amplify suggests that there may be an insertion or other genomic disruption at this location of the chromosome; the insertion of transposable elements is a common cause of spontaneous mutation in the mouse [55]. We extracted DNA from 55 affected mice from the colony, and in all of them this specific sequence failed to amplify, while an adjacent sequence worked. In 56 unaffected mice, both sequences were amplified (Fig. 8e, f; primers in Additional File 2: Table S3).

### Three other lines which underwent exome sequencing

In addition to the above seven lines, mice from three other lines displaying nonsegregating phenotypes were sequenced (MAKN, MATH and MBVF colonies, Fig. 1). The mutation underlying the rapidly progressive hearing loss phenotype seen in the MAKN line was a point mutation in the *S1pr2* gene, named stonedeaf (*S1pr2*^*stdf*^). Mice homozygous for this allele displayed a rapid reduction in endocochlear potential (EP) between P14 and P56 which correlated with the progression of their hearing loss, while hair cell degeneration followed at a later age [12].

In the case of the mutation in the MATH line, we were not able to establish a breeding colony inheriting the phenotype, but we were able to confirm the presence of the *Klhl18*^*lowf*^ mutation from the exome sequence, which is in accordance with the low frequency hearing loss exhibited by these mice (Fig. 1).

The affected mice from the MBVF line displayed variably raised thresholds across all frequencies (Fig. 1); we named the allele variable thresholds, *vthr*. However, because of this extreme variability, we were unable to carry out a backcross or maintain the *vthr* phenotype within the colony. We did observe that the thresholds of 7 affected mice, while variable at 14 weeks old, progressed to more severe hearing loss at 6 months old (Additional File 1: Fig S4c). We collected and examined the middle ears of these affected mice and related unaffected mice at ages over 6 months and did not observe any middle ear defects (*n* = 7 affected, 17 unaffected). Because of this variability in ABR thresholds, we did not restrict the zygosity of identified variants during variant processing (Additional File 2: Table S1). After quality and impact filtering, we found 221 potential high impact variants, including 15 large structural variants that were predicted to affect 30 known deafness genes between them (Additional File 2: Table S2).

### ES Cell sequencing

The 25 lines with spontaneous mutations were derived from several different parental embryonic stem cell lines (Table 1). We carried out whole exome sequencing on three of these lines (JM8.F6, JM8.N4 and JM8.N19) and used Sanger sequencing of selected regions to check two others (JM8A1.N3 and JM8A3.N1) [56]. For the eight alleles affecting hearing described above (including *S1pr2*^*stdf*^), we found that the mutations were not present in the parental ES cell lines (Table 1). The *Klhl18*^*lowf*^ mutation, the only one seen in multiple lines, was not found in any of the ES cell lines from which we obtained sequence (Table 1).

In order to find out whether any variants seen in the mice could have been derived from the parental ES cell lines, we compared the JM8.F6 and JM8.N4 whole exome sequencing to whole exome sequencing from four descendant mice (two from the MCBX line and two from the MATH line, both of which carried the spontaneous *Klhl18*^*lowf*^ mutation, Fig. 1, Table 1). For this, we adapted the variant filtering steps and processed exome sequence data from each mouse independently (Additional File 2: Table S4a). We compared the high-quality variants identified by the different callers and found that a subset of ES cell variants was indeed found in the mice created from each line (e.g. 8 out of 1105 variants called by SAMtools were found in the JM8.F6 ES cells and in both MCBX mice, Additional File 1: Fig. S7). We chose 21 high-quality, high impact variants for confirmation by Sanger sequencing but most variants were not validated in either the ES cells or the mice (Additional File 2: Table S4b). However, two variants were found in both JM8.F6 and the two MCBX mice; one point mutation identified by SAMtools and one small indel called by Dindel (Additional File 2: Table S4b). These variants were not seen in the two MATH mice, which shared the same low frequency hearing loss mutation and the spontaneous *Klhl18*^*lowf*^ allele, nor in the JM8.N4 ES cell sequence.

### Potential sources of the spontaneous mutations affecting hearing

As none of the spontaneous mutations we found that affected hearing came from the original parental ES cell lines (prior to genetic manipulation), we examined the pedigrees of the mice originally found to have these phenotypes and the mice used as founders for our eight breeding colonies. For each line, we identified the latest possible point at which the mutant allele could have arisen, assuming that it only occurred once. In five lines, the mutation could have arisen just two generations before it was observed (MEBJ (*Tkh*), MHER (*spdz*), MEEK (*rhme*), MDLY (*ttch*) and MEWY (*jigl*)). In the MAKN (*stdf*) line, the mutation must have arisen no less than three generations before the phenotype was observed (Additional File 1: Fig. S8). However, in the MFFD (*rthm*) line, the mutation must have been present in the chimaeric offspring of the microinjected founder (Additional File 1: Fig. S8). For the lines bearing the *Klhl18*^*lowf*^ allele, the most likely source is the wildtype colony used for embryo donors, microinjection and colony expansion. We checked the pedigrees of the mice homozygous for the *Klhl18*^*lowf*^ allele and confirmed that in each case there was an ancestral mouse from the same wildtype colony which could have passed on the *Klhl18*^*lowf*^ allele to both the dam and sire of the homozygous mouse. We then constructed pedigrees for these wildtype mice and determined that it is likely that the *Klhl18*^*lowf*^ allele was present in several of the founders of the C57BL/6 N wildtype colony used to expand the mutant lines [9].

**Fig. 9:**
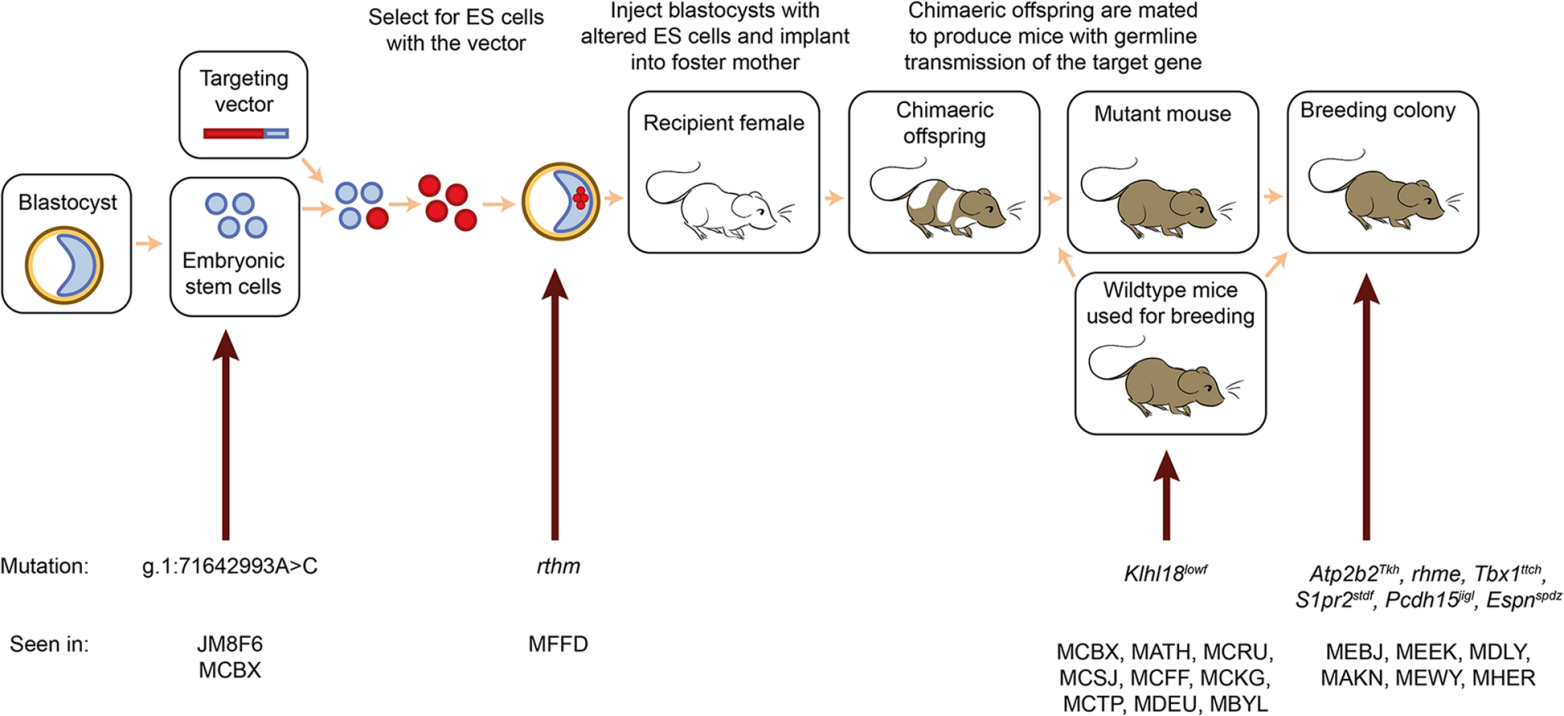
There are many opportunities for spontaneous mutations to arise during the process of making a targeted knockout allele. A schematic showing the stages of making knockout mice. Spontaneous mutations can arise at any point in this process. The mutations described in this paper are shown at the bottom, with arrows indicating the latest possible time at which that mutation could have occurred. If a spontaneous mutation occurs in the cultured embryonic stem cells before targeting, it has the potential to affect multiple mouse lines, but although we detected variants in MCBX mice which were present in the parental ES cell line JM8F6 (eg g.1: 71642993A > C, Additional File 1: Fig S7, Additional File 2: Table S4b), none of the eight mutations affecting hearing were found in any of the parental ES cell lines (Table 1). A mutation arising later in the process may be specific to a single line (such as the *rthm* allele, which is likely to have arisen in the targeted ES cell) or a single mating within that line, which is likely to be the case for most of the mutations described here. The *Klhl18*^*lowf*^ mutation, on the other hand, probably arose within the wildtype line used for expansion for all the colonies in which mice with the low frequency hearing loss phenotype were found

In summary, one mutation was present at the point of microinjection (MFFD, *rthm*) and therefore arose when the ES cells were targeted or during the ES cell processing prior to microinjection, and one is likely to have arisen spontaneously in a wildtype colony (MCBX, *Klhl18*^*lowf*^). The remaining six mutations could have occurred either during ES cell targeting and processing or during breeding of the colony carrying a targeted allele (Fig. 9).

## Discussion

Here we have described seven mutant alleles affecting hearing which, along with the previously described *S1pr2*^*stdf*^ mutation [12], arose as spontaneous mutations within a targeted knockout programme (Table 1). In total, we observed 25 lines with hearing impairment which did not segregate with the targeted allele (Fig. 1), and we identified the causative mutation in 16 of them. It is likely that in the cases where we could not establish a breeding colony carrying the phenotype, the related mice we obtained were not carrying the mutation. However, it is possible that in the lines where only one mouse was found to be affected, the mutation causing hearing loss was a somatic mutation (e.g. MGKQ, Fig. 1). In one case (MBVF), the phenotype was variable and could not be reliably maintained. We have identified six novel alleles of known deafness genes (*Klhl18* [10, 11], *S1pr2* [12, 57–59], *Atp2b2 *[24, 25, 31–36], *Tbx1 *[43, 44, 60, 61], *Pcdh15 *[62] and *Espn *[63]), two of which (*Tbx1*^*ttch*^ (MDLY), *Atp2b2*^*Tkh*^ (MEBJ), Table 1) are hypomorphs which may be useful for in-depth studies of the function of these genes. We also identified four candidate deafness genes (*Ctxn3*, *Ccdc192*, *Map3k5* and *Map7*) within the *rthm* and *rhme* deletions in the MFFD and MEEK lines, and more study is required to identify which gene is responsible for the hearing phenotype or whether, in the case of the *rthm* deletion, it is one of the noncoding RNA genes that underlies the deafness and vestibular dysfunction in this line. For the *rhme* deletion, existing expression data and the associated male infertility support *Map7* as the best candidate gene. Full analysis of these candidates, especially the noncoding RNA genes in the *rthm* deletion, is outside the scope of this study, which aimed to describe the spontaneous mutations arising within the Mouse Genetics Project and to identify their likely origin.

It is likely that over a thousand genes contribute to the development and function of the ear, making hearing impairment relatively sensitive to background mutation rates [9, 11]. From the 9016 mice (2218 wildtype mice and 6798 targeted mutants) screened by ABR in the current study, 55 had a non-segregating phenotype (0.6%). However, seven of the eight mutations causing a hearing phenotype were inherited in a recessive way, so that we had to screen a mouse with two copies to detect it, and only four mice per colony were normally screened by ABR, although in some cases wildtype littermates were included in the control cohort. Thus, the number of new mutations we found causing deafness is likely to be an underestimate, as many colonies harbouring new mutations causing deafness will not have led to a homozygote reaching the screening cohort. Like many phenotypes, hearing impairment in a mouse, when unaccompanied by vestibular dysfunction, is a subtle phenotype and easy to miss if it is not actively investigated. It is highly likely that multiple other mutations which have no impact on hearing nor any gross impact on the appearance, behaviour or viability of the mice exist within all these lines. We found two such mutations in the process of sequencing the MDLY (*ttch*) mice: a missense mutation in *Muc13* and a deletion in the Kabuki syndrome gene *Kmt2d* (also known as *Mll2*), neither of which affected hearing nor had any obvious effect on homozygotes (Additional File 1: Fig. S6).

When we first observed the non-segregating phenotypes, particularly the widespread *Klhl18*^*lowf*^ mutation, we suspected that the mutations arose in the ES cells used to make the mice, either before or after insertion of the manipulated allele. Indeed, a previous missense mutation in *Atp2b2* has been reported to have arisen during clonal expansion of targeted ES cells [34], and multiple other examples of off-target mutations arising in targeted ES cells have been described [64, 65]. However, when we sequenced the parental ES cell lines, we did not find any of these mutations, and from our pedigree analyses, only one can be unambiguously traced to the founder of the line (MFFD, *rthm*), although since we can only identify the latest possible time of occurrence from the pedigrees, we cannot rule out the possibility that the other mutations occurred within the targeted ES cell. In addition, the presence of the *Klhl18*^*lowf*^ mutation in multiple lines suggests that it arose within the wildtype colony used for expansion of the mutant lines (Fig. 9). We therefore suggest that it is likely that at least some of the mutations affecting hearing arose during breeding. Thus, regardless of the method of genome manipulation or how long ago a mutation was made, the potential for spontaneous and off-target mutations to affect a mutant animal being studied must always be borne in mind. In particular, a non-segregating mutation that affects the phenotype under study could result in apparently variable penetrance or expressivity of the phenotype. It is possible that the variable phenotype observed in the MBVF (*vthr*) line (Fig. 1) is the result of more than one spontaneous mutation.

As well as being a reminder to pay attention to unexpected phenotypes, these results highlight the critical importance of proper data collection and retention in any study. The accurate breeding records and careful tracking of the thousands of mice tested in the Mouse Genetics Project were critical to the detection, isolation and identification of these new mutations. Furthermore, confirmation of candidate variants by Sanger sequencing was essential to identify false calls from exome sequence.

## Conclusions

Any process which involves mutagenesis has the potential to introduce unwanted mutations as well as the desired mutant allele. Our results here show that breeding alone can result in mutations which have an observable effect on phenotype and provide a cautionary note for any study involving animals, not just those with targeted mutant alleles. Spontaneous mutations may arise in a ‘wildtype’ strain and remain unnoticed until looked for, as in the *Klhl18*^*lowf*^ mutation. However, such spontaneous mutations can also provide useful alternative alleles, such as the hypomorphic *Tbx1*^*ttch*^ and *Atp2b2*^*Tkh*^ alleles, and suggest candidate disease genes such as *Map7*, and so in addition to a warning, this study also represents the unexpected benefits of a large-scale intensive mutagenesis programme.

## Methods

### Husbandry

Mice were housed in individually ventilated cages (Tecniplast) with Datesand Aspen bedding, with up to 5 adult mice of a single sex in each cage. Extra nesting material and cardboard tubes were provided for environmental enrichment. The temperature and humidity were controlled (21 ± 2 °C, and 55 ± 10%, respectively), and a 12-h light/dark cycle was maintained throughout the study. The mice had free access to water and food (LabDiet PicoLab Rodent Diet 20, St. Louis, MO, USA) and were checked daily for signs of ill health.

### Generation of mice

Mice carrying knockout first conditional-ready alleles were observed for gross behavioural abnormalities using a modified SHIRPA (SmithKline Beecham, Harwell, Imperial College, Royal London Hospital Phenotype Assessment) test at 9 weeks old, and tested by ABR at 14 weeks old, as described in [9] and [11]. Mice assigned to the phenotyping pipeline could not be withdrawn for investigation of these non-segregating phenotypes, so mice from the same line, as closely related to the affected mice as possible, were used to set up colonies to screen for the spontaneous mutation. 25 lines were found to carry a spontaneous mutation involving hearing impairment, and eight new breeding colonies were successfully established with the observed phenotype reliably inherited. All mutations were generated and maintained on the C57BL/6 N background. These eight mouse lines will be available via the European Mouse Mutant Archive. Two further mouse lines were used for complementation testing of compound heterozygotes: *Klhl18*^*tm1a(KOMP)Wtsi*^ [9] and *Tbx1*^*tm1Bld*^ [45].

### Experimental design

Affected mice were compared to unaffected littermate controls of the same age and, where possible, sex, although not to the exclusion of mice of the required phenotype available. Randomisation is not appropriate for experiments with paired mutant and littermate controls. Samples younger than P14 were collected prior to genotyping, effectively blinding the collection. No other blinding was carried out. Sample sizes were calculated using the power calculator at dssresearch.com along with data from previous experiments of the same kind, with a 5% significance level in all cases. Individual power calculations and exclusion criteria are listed under each relevant method; if no exclusion criteria are listed, all experimental mice were included. The *n* numbers for each experiment are given in the figure legends and always refer to the number of mice (and thus also the number of times each experiment was performed).

### Auditory Brainstem Response (ABR)

ABR tests were carried out as previously described [11, 66, 67]. We used a broadband click stimulus and shaped tonebursts at a range of pure tone frequencies at sound levels from 0 to 95 dB SPL, in 5 dB steps. 256 sweeps were carried out per frequency and sound level, and these were averaged to produce the ABR waveform. A stack of response waveforms was used to identify the threshold for each stimulus, which is the lowest intensity at which a waveform could be distinguished.

Power calculation: Six animals per genotype are required for 98.4% power to detect a meaningful effect size of 20 dB given a standard deviation of 8.45 dB.

Exclusion criteria: If a mouse showed evidence of poor physiological condition, such as a reduced heartbeat, during ABR recording, recording was stopped, and the data from that session was not included. This is standard procedure and thus pre-established.

### Assessment of balance

Mouse behaviour was observed in their home cage for signs of circling or headbobbing. To detect balance dysfunction, we also used the contact righting reflex test. Mice were placed in a large Petri dish sized such that the back of the mouse was in contact with the dish lid but not under pressure. The Petri dish was inverted, and the mouse was monitored for 30 s. If it did not turn itself over within that time, it was marked as affected, while if it turned itself over promptly, it was marked as unaffected. Some mice righted themselves after a delay and were tested a second time.

Exclusion criteria: If the results of the righting test were unclear, the mouse was not used for mapping.

### Linkage mapping

For each line, affected males were outcrossed to C3HeB/FeJ females. For the lines displaying recessive inheritance, the offspring were backcrossed to affected mice of the same line. For the single line displaying semidominant inheritance (MEBJ, *Atp2b2*^*Tkh*^), the outcross offspring were backcrossed to C3HeB/FeJ wildtype mice. Backcross offspring were assessed by ABR or contact righting reflex, then after culling, a tissue sample was collected for DNA extraction. The initial genome scan was carried out using a standard marker panel (Additional File 2: Table S5), then once the linkage region was established, it was narrowed down using more backcross mice, more markers within the region and, where necessary, strain-specific SNVs (Additional File 2: Table S5).

### Exome sequencing

Two affected mice of each line were selected for exome sequencing. DNA was extracted using phenol and chloroform, and exome sequencing was carried out by the Wellcome Trust Sanger Institute (WTSI), with the exception of the *spdz* sequencing, which was carried out by Novogene (Hong Kong). Genomic DNA (approximately 1 ug) was fragmented to an average size of 150 bp (WTSI) or 180–280 bp (Novogene) and subjected to DNA library creation using the Agilent SureSelect Mouse All Exon Kit. Adapter-ligated libraries were amplified and indexed via PCR. Enriched libraries were subjected to 75 bp paired-end sequencing (HiSeq 2000; Illumina), with the exception of the *spdz* libraries, which were subjected to 150 bp paired-end sequencing (HiSeq 4000; Illumina) following the manufacturer’s instructions. Sequences were aligned to GRCm38 using bwa [68], with the exception of the *spdz* fastq files, which were checked and processed using FastQC [69] and Trimmomatic [70], then aligned to GRCm38 using hisat2 [71]. All bam files were improved by local realignment around insertions and deletions discovered in the mouse genomes project [72] using GATK [73] (Additional File 2: Table S6). Bam files were processed using Picard and Samtools [74, 75]. Raw data can be downloaded from the European Nucleotide Archive, studies PRJEB2585, PRJEB5221 and PRJEB45713. Individual sample accession numbers are in Additional File 2: Table S6c.

### Variant identification and investigation

Variant calling was carried out using Samtools, Dindel and Pindel [75–78], and we also used BreakDancer to detect any large structural variants with breakpoints within the exome [77] (Additional File 2: Table S6). Variants were annotated using the Ensembl Variant Effect Predictor [79], and filtered by quality, by segregation and by presence in other lines, including known variants from the Ensembl database and the Mouse Genomes Project [72, 80, 81] (Additional File 1: Table S1). For the ES cells and the MBVF line, we could not use mapping cross information so quality and impact filtering were carried out instead as the final step (Additional File 2: Table S6). IGV [82] was used to check for large deletions in the critical region. Candidate variants were first confirmed by Sanger sequencing, then checked for segregation with the phenotype across the colony as well as all backcross mice (Additional File 2: Table S3). Protein modelling was carried out using Phyre2 [15] to identify a suitable protein model and Pymol [83] to view the model and highlight variant residues. All Sanger sequencing was carried out by Source Bioscience and analysed using Gap4 [84]. Venn diagrams were generated using the online Bioinformatics and Evolutionary Genomics tool [85].

### Gene expression analysis using the gEAR

To investigate candidate gene expression, we used single-cell RNAseq data from the mouse inner ear at E16, P1, P7 [86, 87], P15 [88, 89], P20 [90, 91] and P30 [92, 93], accessed via the gEAR portal [40, 94]. Expression levels were normalised to *Hprt* expression. Ten marker genes were chosen for comparison (*Myo7a* for hair cells, *Fgf8* for inner hair cells, *Slc26a5* for outer hair cells, *Sox2* for non-sensory cells, *S100b* for inner pillar cells, *Hes5* for Deiters’ cells, *Kcne1* for marginal cells, *Kcnj10* for intermediate cells, *Epyc* for root cells and *Anxa1* for spindle cells). Of the ten candidate genes (including four protein-coding genes, four lncRNA genes, one miRNA and one snRNA), only *Ctxn3*, *Map7* and *Map3k5* had sufficient expression data in the gEAR.

### Scanning electron microscopy (SEM)

The temporal bones were isolated. The inner ears were dissected out and fixed by 2.5% glutaraldehyde in 0.1 M sodium cacodylate buffer with 3mM calcium chloride at room temperature for 3 h. Cochleae and the vestibular system were finely dissected in PBS. This was followed by further processing using an osmium-thiocarbohydrazide-osmium (OTOTO) method [95]. The samples were dehydrated in increasing concentrations of ethanol, critical-point dried (Bal-Tec CPD030), mounted and examined under a HITACHI S-4800 or a JEOL JSM 7800F Prime Schottky field emission scanning electron microscope (for *Pcdh*^*jigl*^ and *Espn*^*spdz*^ mice respectively). Images of the organ of Corti were taken at roughly 20% intervals along the cochlear duct and the macula of the utricle and saccule and crista of the ampullae were also imaged. Whole images were adjusted in Photoshop to normalise dynamic range across all panels.

Exclusion criteria: If dissection damage was too great to observe hair cells in either cochlea, the mouse was not counted. This is standard procedure and thus pre-established.

### Middle ear dissection, inner ear clearing and microCT scanning

After culling, the ear canals were checked for cerumen, then the mouse was decapitated and the bulla, tympanic membrane and middle ear cavity inspected for any abnormalities, including the presence of fluid, inflammation or any other obstructions in the middle ear. Observations were recorded on a standard tick sheet. Ossicles were dissected out and stored in 10% formalin. The inner ear was removed and fixed in Bodian’s fixative (75% ethanol, 5% acetic acid, 5% formalin, in water), washed in water and 70% ethanol, then cleared by gentle rotation in 3% KOH for 3 days, changed daily. Samples were then placed in G:E:B (glycerol, 70% ethanol and benzyl alcohol, mixed in a ratio of 2:2:1 by volume) for the final stage of clearing, then stored in G:E (glycerol and 70% ethanol in equal volumes). Images of ossicles and middle ears were taken using a Leica stereomicroscope with a Leica DFC490 camera. To carry out microCT scans, cochleae were immobilised using cotton gauze and scanned with a Scanco microCT 50 to produce 14μm voxel size volumes, using an X-ray tube voltage of 80kVp and a tube current of 80μA. An aluminium filter (0.05 mm) was used to adjust the energy distribution of the X-ray source. To ensure scan consistency, a calibration phantom of known geometry (a dense cylinder) was positioned within the field of acquisition for each scan. Test reconstructions on this object were carried out to determine the optimum conditions for reconstruction, ensuring consistency in image quality, and minimising blurring. Reconstruction of the cochlea was performed in Thermo Scientific Amira software.

### Immunohistochemistry and trichrome staining

Samples from adult mice were collected, fixed in 10% formalin, decalcified in 0.1 M Ethylenediaminetetraacetic acid (EDTA), embedded in paraffin wax and cut into 8μm sections. Samples from P4 pups were treated similarly, but no decalcification step was needed. For histological analysis, slides were stained using a trichrome stain, containing Alcian blue, Sirius red and Haematoxylin. Immunohistochemistry was carried out using a Ventana Discovery machine and reagents according to the manufacturer’s instructions (DABMap™ Kit (cat.no 760–124), Haematoxylin (cat.no 760–2021), Bluing reagent (cat.no 760–2037), CC1 (cat.no 950–124), EZPrep (cat.no 950–100), LCS (cat.no 650–010), RiboWash (cat.no 760–105), Reaction Buffer (cat.no 95–300) and RiboCC (cat.no 760–107)). Primary antibodies used were rabbit anti-PMCA2 (Abcam, cat. no: ab3529, RRID:AB_303878, diluted 1:500), rabbit anti-MUC13 (Abcam, cat. no: ab124654, RRID:AB_11129750, diluted 1:50) and rabbit anti-MYO7A (Proteus, cat. no: PTS-25–6790, RRID:AB_10015251, diluted 1:100), and the secondary antibody was anti-rabbit (Jackson ImmunoResearch, cat.no 711–065-152, RRID:AB_2340593, diluted 1:100). All antibodies were validated by the manufacturer and/or had been successfully used for immunohistochemistry on paraffin-embedded sections of mouse tissue in previous studies [96, 97]. Antibodies were diluted in staining solution (10% foetal calf serum, 0.1% Triton, 2% BSA and 0.5% sodium azide in PBS). A Zeiss Axioskop 2 microscope was used to examine slides, and photos were taken using a Zeiss Axiocam camera and the associated Axiocam software. Images were processed in Adobe Photoshop; minimal adjustments were made, including rotation and resizing. Where image settings were altered, the adjustment was applied equally to affected and unaffected samples and to the whole image. Variation in staining intensity between sections was minor, and there was no variation in staining location.

Exclusion criteria: If damage which occurred during sectioning and staining was too great to observe hair cells, the sample was not counted. This is standard procedure and thus pre-established. Only one set of sections from a pair of *Espn*^*spdz*^ mice were excluded for this reason and have not been counted in the total assessed.

### RNA extraction, RTPCR and qPCR

We collected the brains of adult *Espn*^*spdz*^ (P25) and *rthm* (P28) mice, which were snap-frozen in liquid nitrogen, and the organs of Corti of 4-day-old (P4) *Atp2b2*^*Tkh*^ mice, which were dissected out and stored at − 20 °C in RNAlater stabilisation reagent (Ambion). All RNA dissections were carried out during a fixed time window to avoid circadian variation (*Espn*^*spdz*^: between 2.5 and 3.5 h after lights on; *rthm* and *Atp2b2*^*Tkh*^: between 6 and 7.5 h after lights on). For the brains, RNA was extracted using TRIzol, in some cases followed by processing through Direct-zol minipreps (Zymo Research, cat. no R2050). For the organs of Corti, RNA was extracted using either QIAshredder columns (QIAgen, cat. no. 79654) and the RNeasy mini kit (QIAgen, cat. no. 74104), or the Lexogen SPLIT kit (Lexogen, cat. no. 008.48), following the manufacturer’s instructions. RNA concentration was measured using a nanodrop spectrophotometer (ND-8000). RNA was normalised to the same concentration within each litter, then treated with DNAse 1 (Sigma, cat. no: AMPD1) before cDNA creation. cDNA was made using Superscript II Reverse Transcriptase (Invitrogen, cat. no: 11904–018) or Precision Reverse Transcription Premix (PrimerDesign, cat. no: RT-premix2). Primers for sequencing cDNA for testing *Espn* splicing in *Espn*^*spdz*^ mice were designed using Primer3 [98] (Additional File 2: Table S3). Quantitative RT-PCR on cDNA from *rthm* and *Tkh* mice was carried out on a CFX Connect qPCR machine (Bio-Rad), using probes from Applied Biosystems (Hprt, cat. no: Mm01318747_g1; Jag1, cat. no: Mm01270190_m1; Atp2b2, cat. no: Mm01184578_m1; Slc12a2, cat no: Mm00436563_m1) and Sso-Advanced Master Mix (Bio-Rad, cat. no: 1725281). Relative expression levels were calculated using the 2^−ΔΔct^ equation [99], with *Hprt* as an internal control. *Jag1* was used to check for the quantity of sensory tissue present in the organ of Corti samples because it is expressed in supporting cells [100, 101]. At least three technical replicates of each sample were carried out for each reaction. Due to the nature of the 2^−ddCt^ calculation, there are always unequal variances between wildtype and mutant groups, and we therefore chose suitable statistical tests; the Wilcoxon rank sum test (Mann–Whitney *U* test, two-tailed) [102] for comparisons between *rthm* wildtypes and homozygotes, and a Welch’s one-way ANOVA [103] for the *Atp2b2*^*Tkh*^ qPCR, which involved three groups (wildtype, heterozygote and homozygote).

#### Power calculations

We estimated the power to detect a difference of 40% for a sample size of 4 wildtypes and 4 homozygotes, with a standard deviation of 0.01 for wildtypes and 0.2 for homozygotes, which is based on previous data (the discrepancy in standard deviation is the result of the 2^−ΔΔct^ calculation of relative expression levels). The power is 99.1%.

#### Exclusion criteria

Data from *Atp2b2*^*Tkh*^ organs of Corti where the *Jag1* levels differed by more than 20% between the samples were not included. This is a pre-established criterion based on our previous work.

## Supplementary Information

**Additional file 1.**

**Additional file 2.**

**Additional file 3.**

## Data Availability

All data generated and/or analysed during this study are included in this published article and supplementary information files, with the exception of the exome sequence data, which are available in the European Nucleotide Archive repository (https://www.ebi.ac.uk/ena/browser/view/PRJEB5221, https://www.ebi.ac.uk/ena/browser/view/PRJEB2585, https://www.ebi.ac.uk/ena/browser/view/PRJEB45713), and the single cell RNAseq data, which are available in the GEO repository (https://identifiers.org/geo:GSE181454, https://identifiers.org/geo:GSE114157, https://identifiers.org/geo:GSE136196, https://identifiers.org/geo:GSE137299) [86, 88, 90, 92]. Data underlying the plots in Figs. 1, 2, 3, 4, 5, 6, 7, 8, S1, S4, S5, S6 and S9 are included in their entirety in Additional File 3. Mutant mouse lines will be available from EMMA.

## Abbreviations

ABR: Auditory Brainstem Response
E16: Embryonic day 16
EDTA: Ethylenediaminetetraacetic acid
ES cells: Embryonic stem cells
EUCOMM: European Conditional Mouse Mutagenesis programme
GWAS: Genome-Wide Association Study
IGV: Integrative Genomics Viewer
KOMP: Knock Out Mouse Programme
OTOTO: Osmium-thiocarbohydrazide-osmium
P4: Postnatal day 4
qPCR: Quantitative polymerase chain reaction
RNAseq: RNA sequencing
SHIRPA: SmithKline Beecham, Harwell, Imperial College, Royal London Hospital Phenotype Assessment
SNV: Single-nucleotide variant
