# Supplementary material for: Identification and characterisation of spontaneous mutations causing deafness from a targeted knockout programme
Source: BMC Biol. 2022 Mar 17;20:67. doi: 10.1186/s12915-022-01257-8 (PMC8928630; doi:10.1186/s12915-022-01257-8)
Supplement: Supplementary file 1 — Additional file 1. [file 12915_2022_1257_MOESM1_ESM.pdf]

ADDITIONAL FILE 1

Identification and characterisation of spontaneous mutations causing deafness from a targeted knockout programme

Morag A. Lewis[1,2*], Neil J. Ingham[1,2] , Jing Chen[1,2], Selina Pearson[2], Francesca Di Domenico[1], Sohinder Rekhi[1], Rochelle Allen[1], Matthew Drake[1], Annelore Willaert[3], Victoria Rook[1], Johanna Pass[1,2], Thomas Keane[2], David J. Adams[2], Abigail S. Tucker[4], Jacqueline K. White[2], Karen P. Steel[1,2]

1. Wolfson Centre for Age-Related Diseases, King's College London, London SE1 1UL
2. Wellcome Sanger Institute, Hinxton, CB10 1SA
3. Research Group of Experimental Oto-rhino-laryngology, Department of Neurosciences, KU Leuven – University of Leuven, Leuven, Belgium
4. Centre for Craniofacial and Regenerative Biology, King's College London, London SE1 9RT

* Corresponding author: Morag.lewis@kcl.ac.uk

a

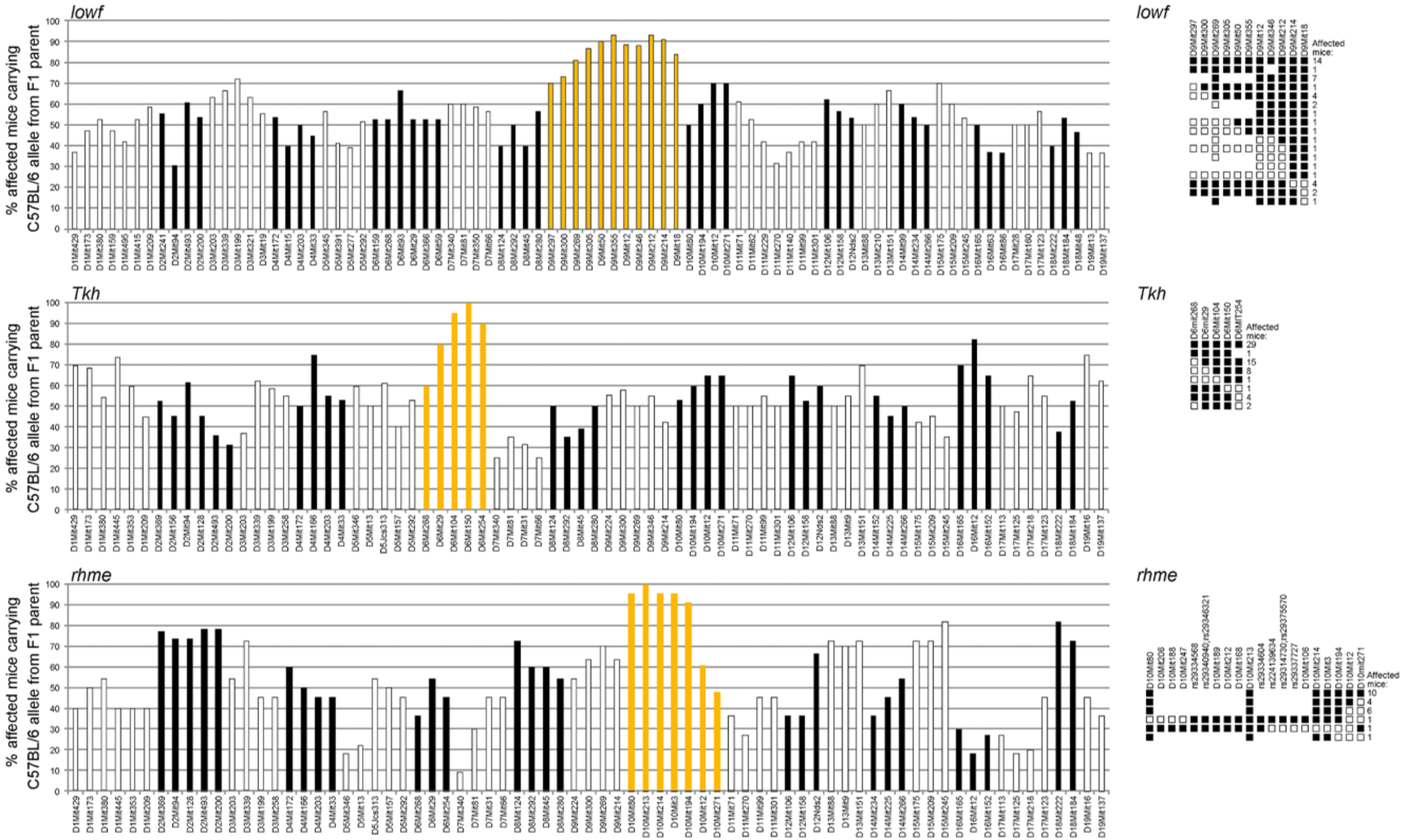

lowf
% affected mice carrying C57BL/6 allele from F1 parent
Tkh
% affected mice carrying C57BL/6 allele from F1 parent
rhme
% affected mice carrying C57BL/6 allele from F1 parent
lowf
Affected mice:
Tkh
Affected mice:
rhme
Affected mice:

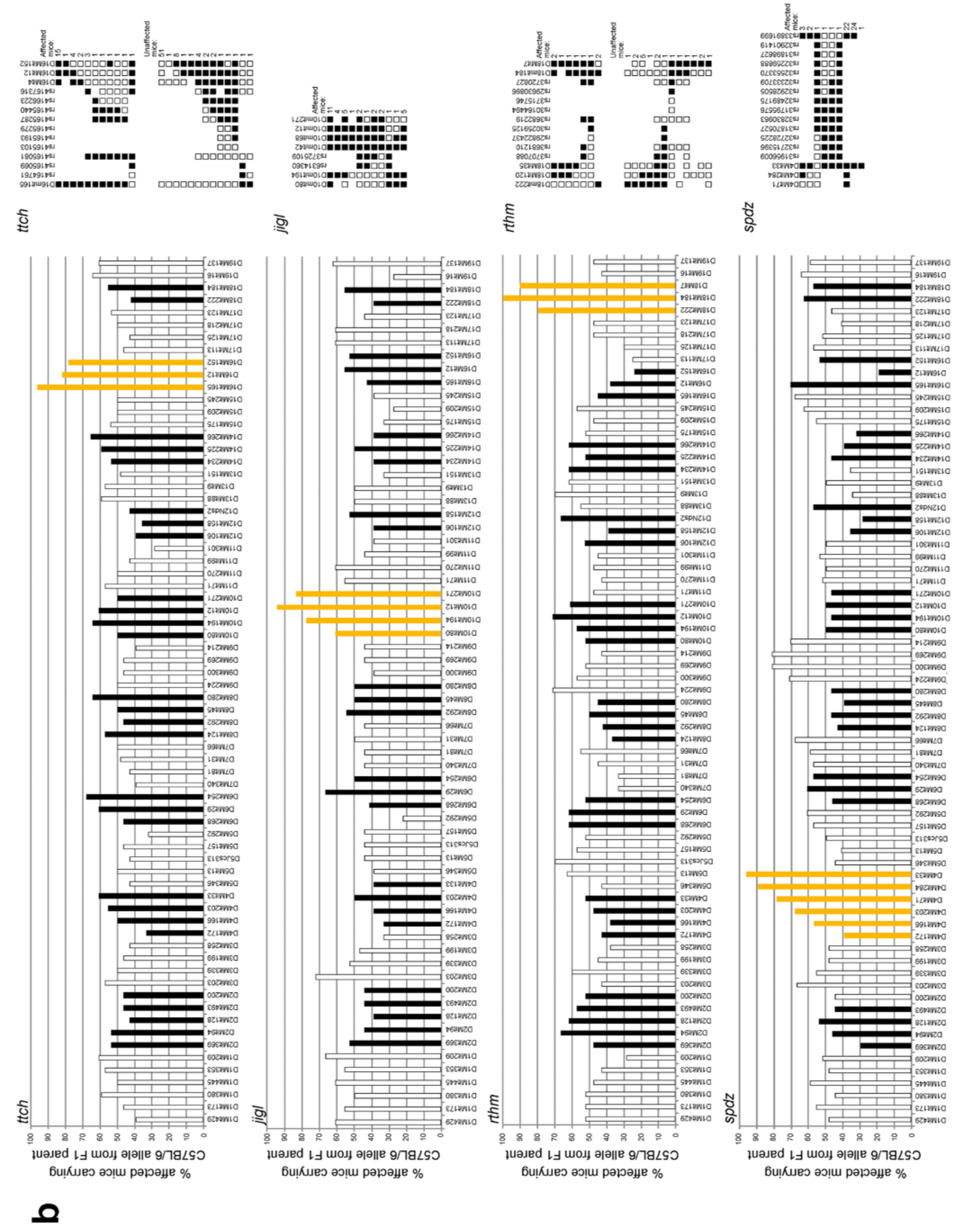

Fig. S1. Genome scans and fine mapping.

The bar charts show the percentage of affected mice carrying the C57BL/6N allele on the chromosome derived from the outcrossed parent at each marker. Alternate chromosomes are indicated by different shading, and the linked chromosome for each mutation is coloured gold. Following the initial mapping, the critical region was identified using more markers and, where necessary, SNVs known to differ between C57BL/6N and C3HeB/FeJ, the two strains used for the mapping crosses. On the right, the haplotype charts show the fine mapping of each allele. Each row represents the marker pattern for the chromosome derived from the F1 parent, with white boxes representing the C3HeB/FeJ-like marker types and black boxes representing the C57BL/6N-like marker types. A change from black boxes to white or vice versa indicates that a recombination event has occurred during meiosis leading to the production of that chromosome in the F1 parent. For all alleles except *Tkh*, the F1 parent was backcrossed to the affected parental line to obtain unaffected mice heterozygous for the causative mutation and affected homozygotes, but because the *Tkh* allele was semidominant, the F1 parent was backcrossed to C3HeB/FeJ, resulting in affected mice heterozygous for the causative mutation and unaffected wildtypes. The number to the right of each row indicates the number of mice showing that particular pattern of marker types. **a** Mapping for *lowf*, *Tkh* and *rhme*. Numbers: *lowf*: n=30 affected mice for the genome scan, 44 affected mice for fine mapping; *Tkh*: n = 20 affected mice for the genome scan, 61 affected mice for fine mapping; *rhme*: n = 11 affected mice for the genome scan, 23 affected mice for fine mapping. **b** Mapping for *ttch*, *jigl*, *rthm* and *spdz*. Numbers: *ttch*: n = 28 affected mice for the genome scan, 31 affected and 75 unaffected mice for fine mapping; *jigl*: n = 18 affected mice for the genome scan, 35 affected mice for fine mapping; *rthm*: n = 21 affected mice for the genome scan, 9 affected and 19 unaffected mice for fine mapping; *spdz*: n = 28 affected mice for the genome scan, 56 affected mice for fine mapping. Data underlying these plots are in Additional File 3.

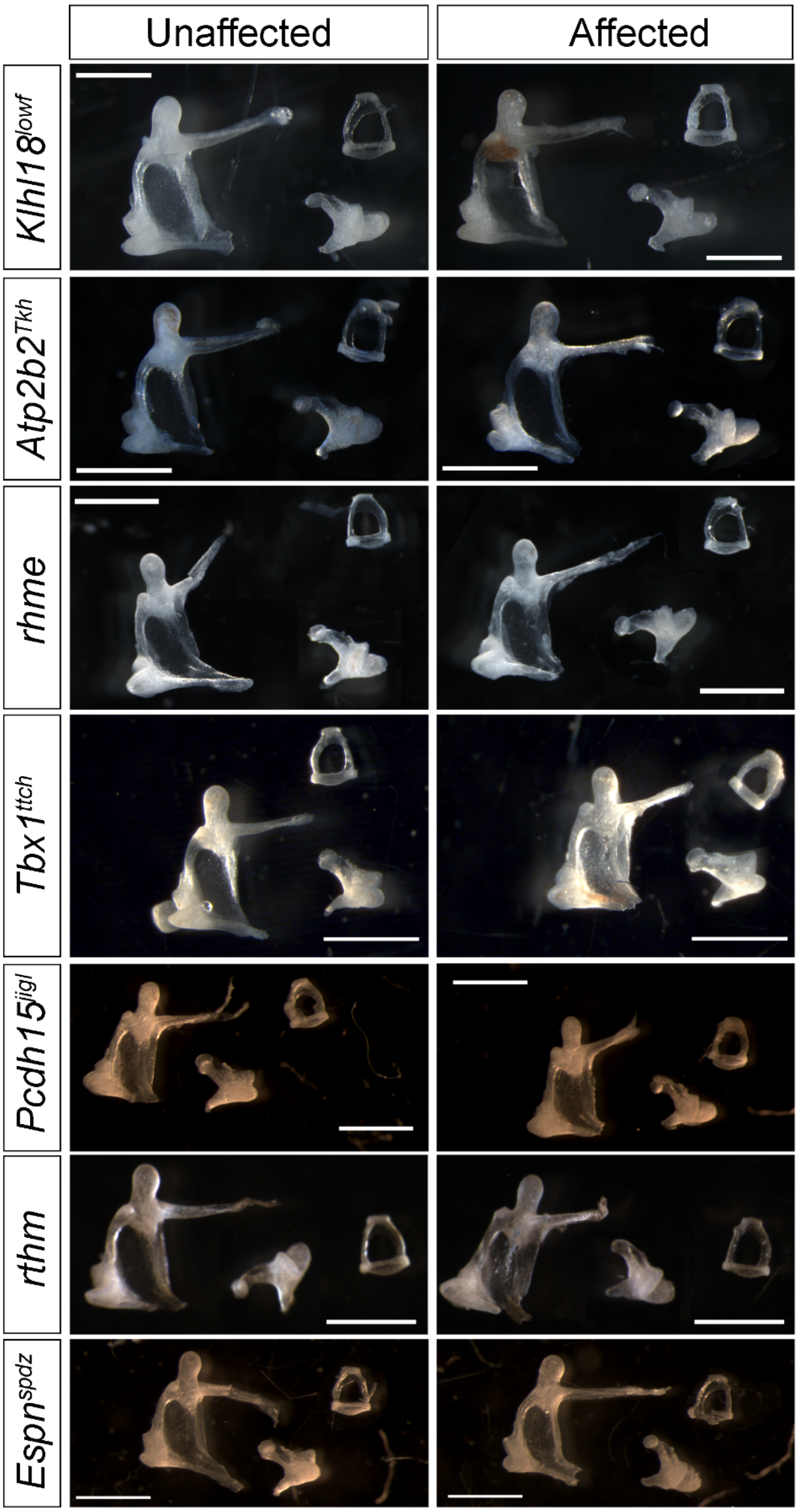

Fig. S2. Middle ear ossicles from mice displaying hearing loss show no defects.

Ossicles from affected mice from all seven lines (right) and unaffected littermates (left). Numbers: *Klhl18*^{lowf} n=5 unaffected, 9 affected mice at P94±1day; *Atp2b2*^{Tkh} n=3 unaffected (wildtype), 3 affected (homozygote) mice at P36±2 days; *rhme* n=3 unaffected, 3 affected mice at P69; *Tbx1*^{ttch} n=3 affected, 3 unaffected mice at P28 or older; *Pcdh15*^{jigl} n=7 affected, 4 unaffected mice at P30±1 day; *rthm* n=3 affected, 3 unaffected mice at P28±1 day; *Espn*^{spdz} n=5 affected, 4 unaffected mice at P57 or older. Scale bar = 1mm. For each panel, the malleus is shown on the left, the stapes on the top right and the incus on the bottom right.

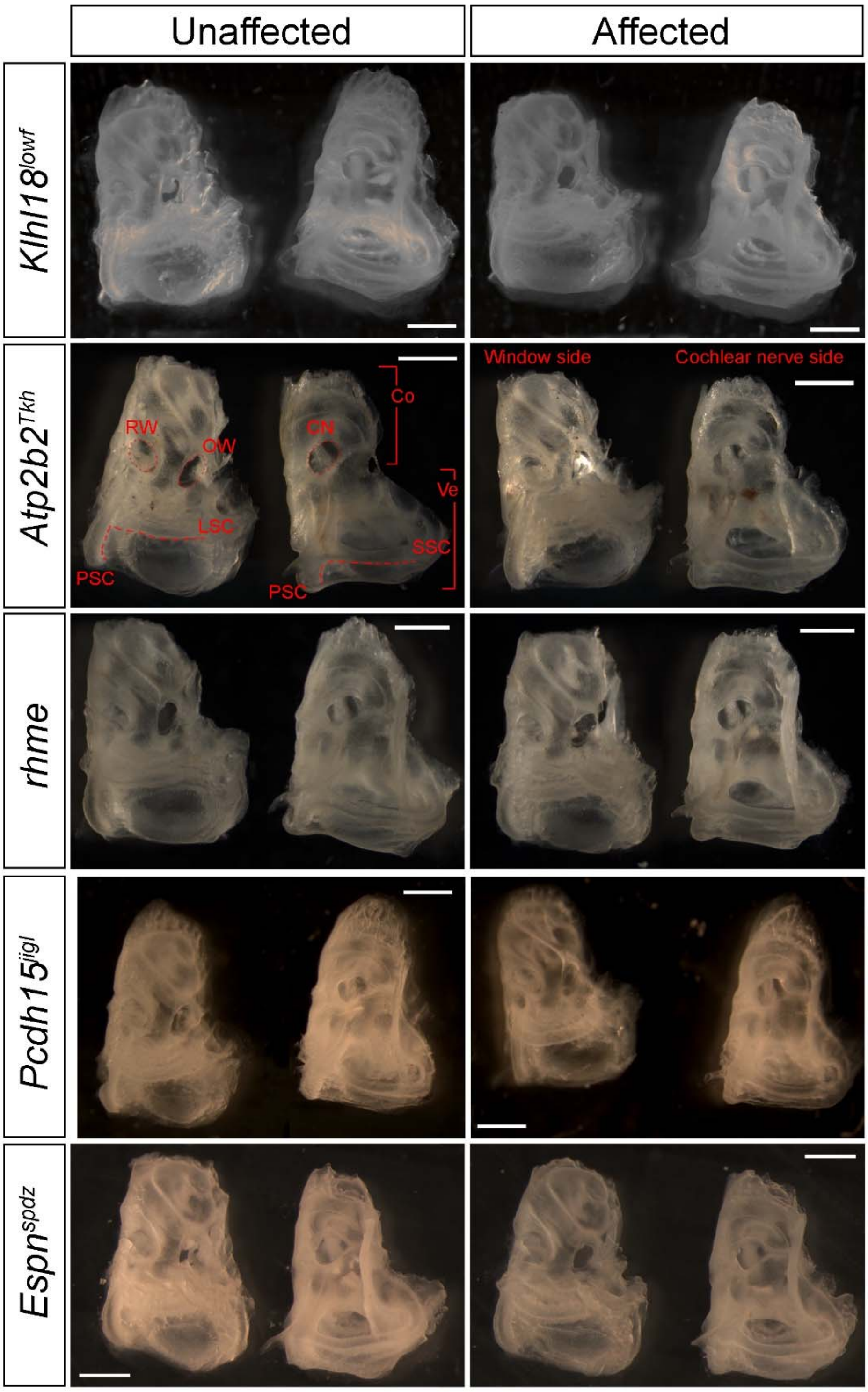

Fig. S3. Cleared inner ears from mice displaying hearing loss which show no gross defects.

Cleared inner ears from affected mice carrying the *Klhl18*^{lowf}, *Atp2b2*^{Tkh}, *rhme*, *Pcdh15*^{jigl} and *Espn*^{spdz} alleles (right) and unaffected littermates (left). Numbers: *Klhl18*^{lowf} n=5 unaffected, 7 affected mice at P43±1day; *Atp2b2*^{Tkh} n=3 unaffected (wildtype), 3 affected (homozygote) mice at P36±2 days; *rhme* n=3 unaffected, 3 affected mice at P69; *Pcdh15*^{jigl} n=12 affected, 8 unaffected mice at P30±1 day; *Espn*^{spdz} n=5 affected, 4 unaffected mice at P57 or older. Scale bar = 1mm. Labels have been added to the *Atp2b2*^{Tkh} inner ears. The middle ear side, with the round (RW) and oval (OW) windows is shown on the left of each panel, and the brain side, where the cochlear nerve exits (CN), on the right. The round and oval windows and the cochlear nerve exits are marked by dotted lines, and the semicircular canals by dashed lines. Brackets indicate the cochlea (Co) and vestibular region (Ve). LSC=lateral semicircular canal; SSC=superior semicircular canal; PSC=posterior semicircular canal.

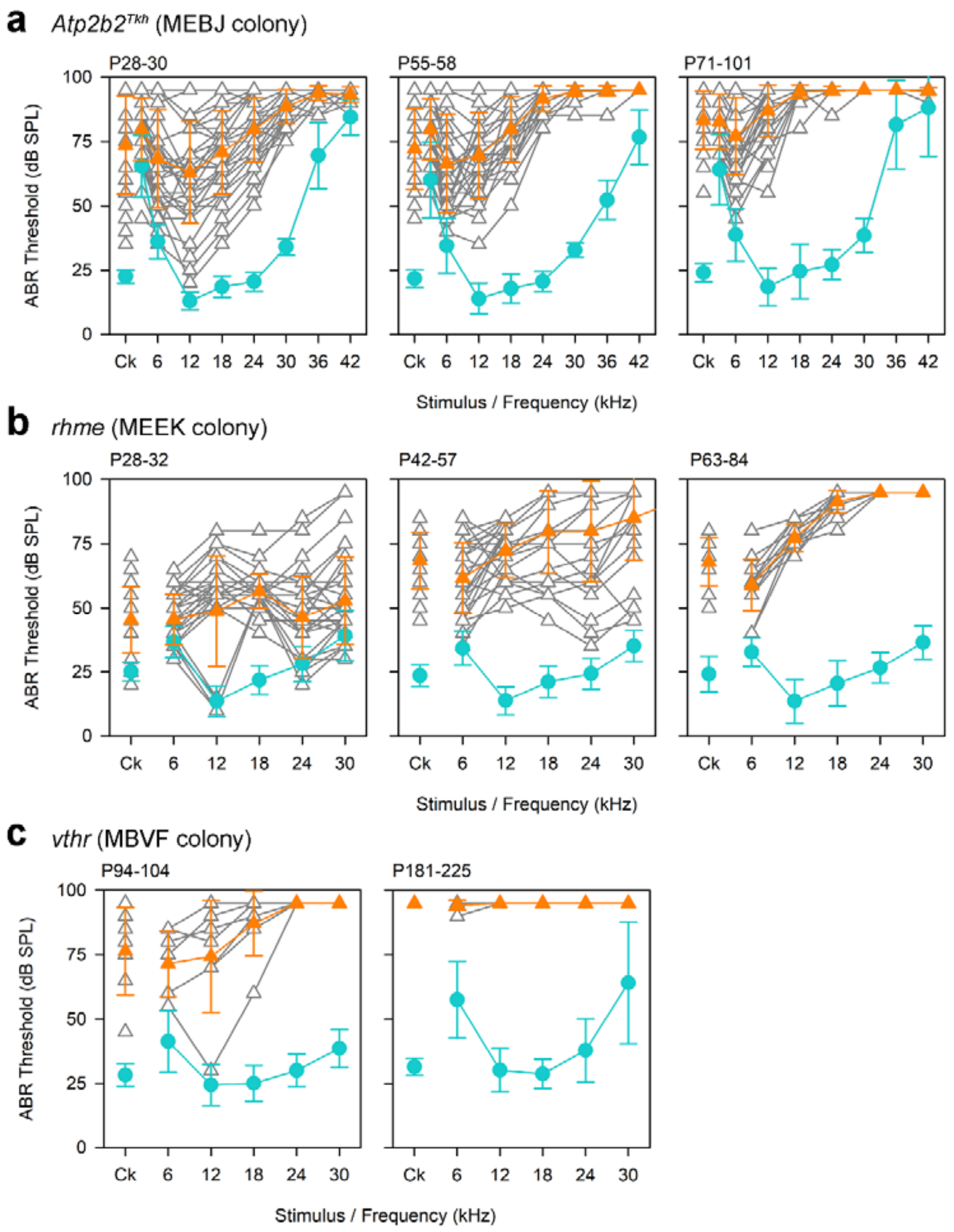

Fig.S4. ABR thresholds of mice from the MEBJ, MEEK and MBVF lines showing progressive division into affected and unaffected mice by threshold.

This figure shows ABR thresholds from mice from the MEBJ (a), MEEK (b) and MBVF (c) colonies, showing both the progression of hearing loss with age and the phenotypic division into affected and unaffected mice. These graphs include both genotyped mice (which will also be represented in other figures) and those mice tested before genotyping was possible. Where possible, the same mice were tested at each age. All error bars are standard deviations. **a** Mean ABR thresholds of affected MEBJ mice carrying the *Atp2b2*^{Tkh} allele and unaffected littermates, showing the progression of hearing loss in affected mice (orange triangles) compared to unaffected mice (teal circles) (n = 10 unaffected and 37 affected at P28-30; n=9 unaffected and 31 affected at P55-58; n=19 unaffected and 37 affected at P71-101). Individual audiograms from affected mice are shown in grey. **b** Mean ABR thresholds from affected (orange triangles) and unaffected (teal circles) *rhme* (MEEK) mice at P28-32 (n = 42 unaffected, 35 affected), P42-57 (n = 83 unaffected, 28 affected) and P63-84 (n = 28 unaffected, 15 affected). Traces from individual affected mice are shown in grey. **c** Mean ABR thresholds of seven MBVF mice carrying the unknown *vthr* mutation at P94-104 and unaffected mice from the same colony (n=7 affected, 89 unaffected) and P181-225 (n=7 affected, 28 unaffected). The hearing loss appears to be variable at the younger age but progresses to be more severe at ages over 6 months. Traces from individual affected mice are shown in grey. Data underlying these plots are in Additional File 3.

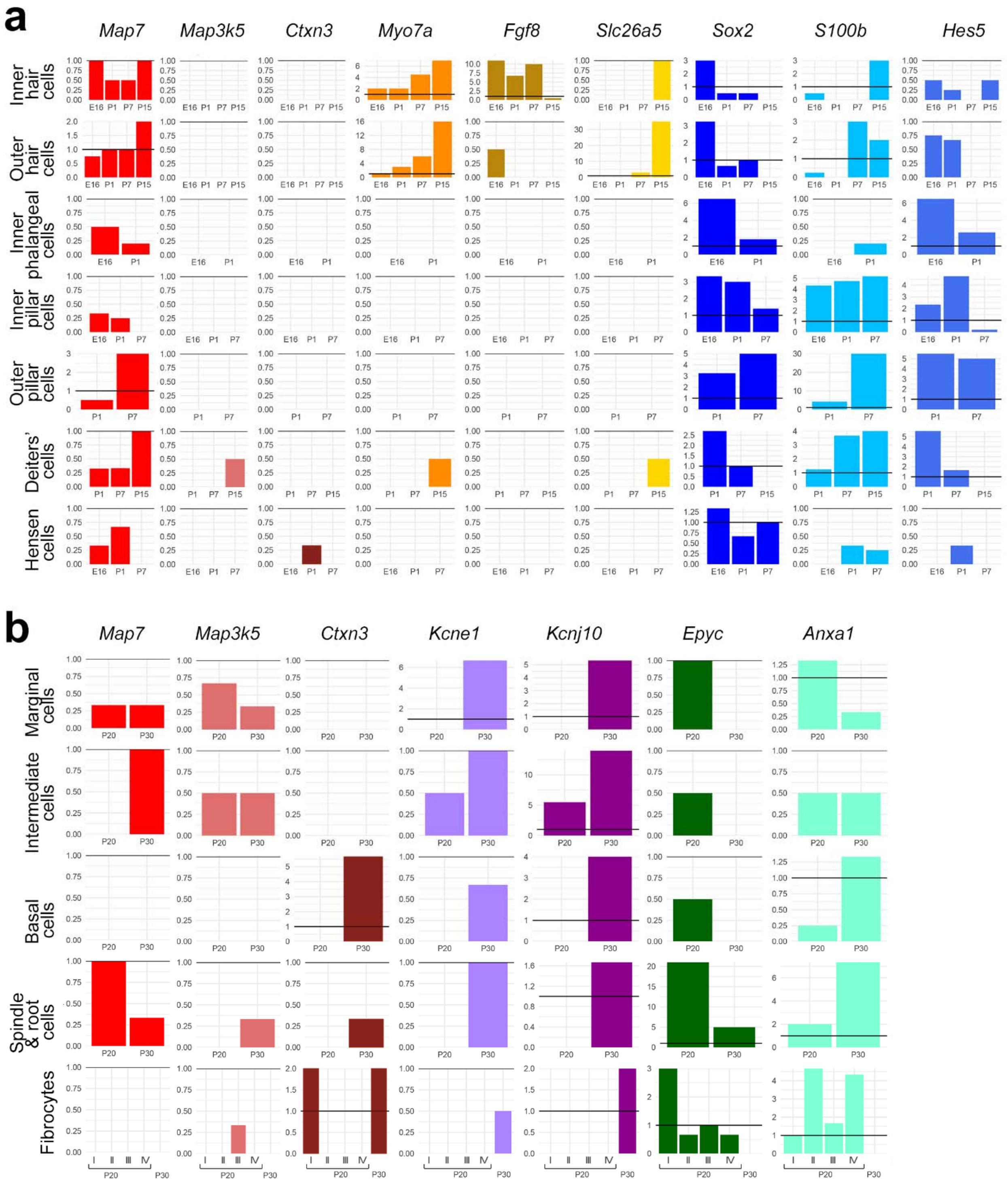

Fig. S5. Expression of candidate genes in different cochlear cell types.

a Expression of candidate genes compared to known marker genes at stages from E16 to P15 in the mouse organ of Corti. **b** Expression of candidate genes compared to known marker genes at P20 and P30 in the mouse lateral wall. Single cell RNAseq data was obtained from the gEAR portal [38, 92] (E16, P1, P7[84, 85], P15[86, 87], P20[88,89], P30[90, 91]). Expression levels were normalised to *Hprt* expression, indicated by a horizontal line at y=1 on each plot. Chosen marker genes were *Myo7a* (hair cells), *Fgf8* (inner hair cells), *Slc26a5* (outer hair cells), *Sox2* (non-sensory cells), *S100b* (inner pillar cells), *Hes5* (Deiters' cells), *Kcne1* (marginal cells), *Kcnj10* (intermediate cells), *Epyc* (root cells), and *Anxa1* (spindle cells). Data underlying these plots are in Additional File 3.

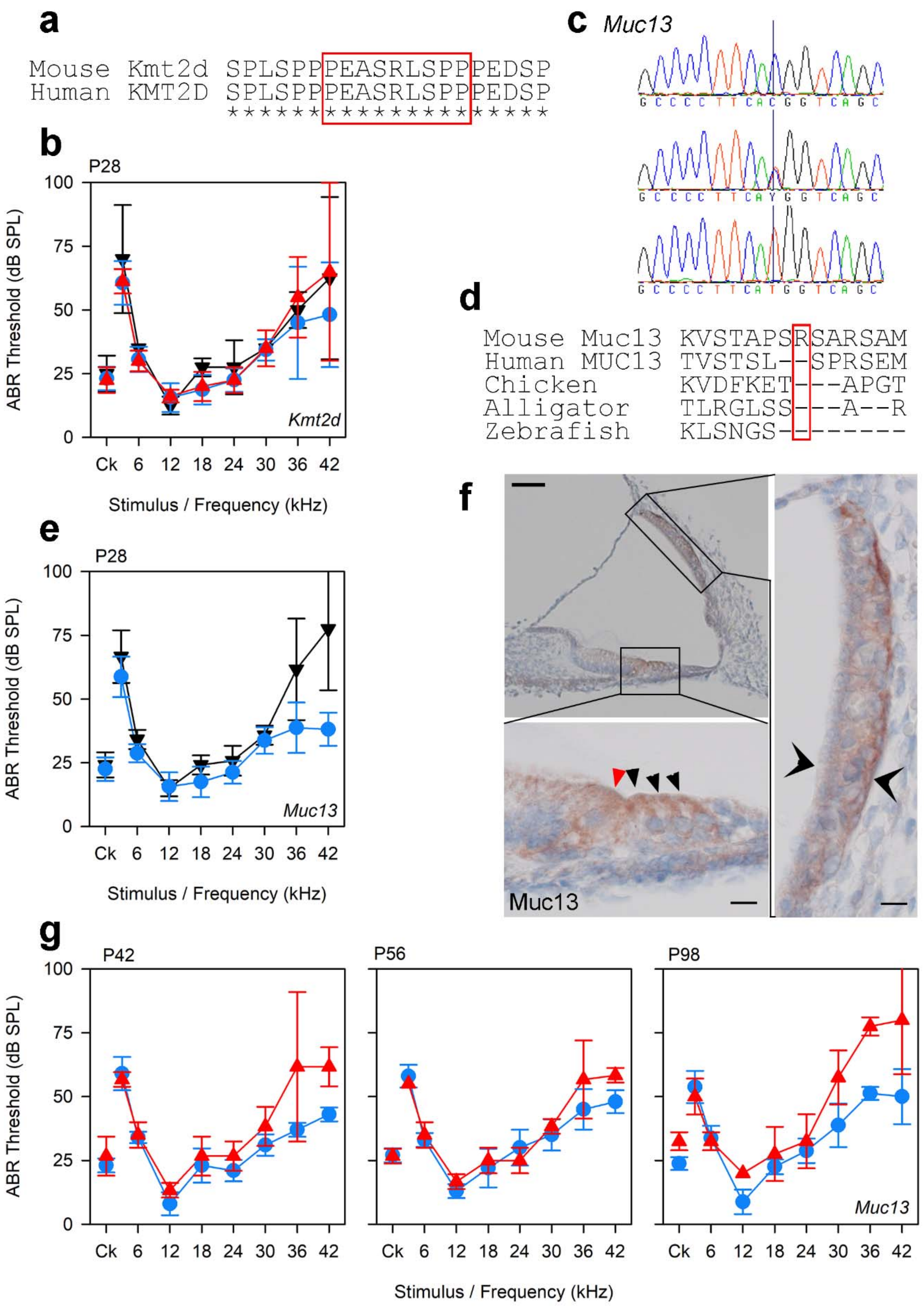

Fig. 6. Two other mutations found in the MDLY line carrying the *ttch* allele did not affect hearing.

a Clustal alignment showing deleted amino acids in *Kmt2d* (red box) caused by the g.15:98863687_98863713del mutation, resulting in a loss of 9 amino acids (ENSMUST00000023741, p.586-594del). The region is conserved between mouse and human but not in other vertebrates checked. **b** Mean ABR thresholds for P28 mice homozygous (n=4, red triangles), heterozygous (n=8,

blue circles) or wildtype (n=2, black inverted triangles) for the *Kmt2d* deletion. All these mice were heterozygous or wildtype for the *Tbx1*^{ttch} allele, and mice homozygous for the deletion have no difference in their ABR thresholds. Error bars are standard deviations. See Additional File 1: Fig. S9d for individual thresholds. **c** Sequence trace showing the missense mutation in *Muc13* (g.16:33807881C>T). **d** Clustal alignment showing the affected amino acid in *Muc13* (red box; p.(Arg334Trp), ENSMUST00000115044). It is not conserved in any other vertebrates checked. **e** Mean ABR thresholds for P28 mice heterozygous (n=8, blue circles) or wildtype (n=6, black inverted triangles) for the *Muc13* missense mutation. All these mice were heterozygous or wildtype for the *Tbx1*^{ttch} allele. Error bars are standard deviations. **f** MUC13 expression at P4 in a mouse heterozygous for both the *Muc13* missense variant and the *Tbx1*^{ttch} allele (n=3). Brown indicates the presence of MUC13 protein, which is visible in the hair cells (arrowheads; red points to the inner hair cell and black to the outer hair cells) and in the basal cells of the stria vascularis (twin open arrowheads). Scale bar = 50µm in the top left panel and 10µm in the two high magnification panels. The section shown is from the region 50% of the distance along the organ of Corti from base to apex. **g** Mean ABR thresholds for mice homozygous (red triangles) or heterozygous (blue circles) for the *Muc13* mutation at later ages (P42 and P56: n=5 heterozygotes, 3 homozygotes; P98 n=4 heterozygotes, 2 homozygotes). All these mice were heterozygous or wildtype for the *Tbx1*^{ttch} allele. Error bars are standard deviations. See Additional File 1: Fig. S9e for individual thresholds. Data underlying plots in this figure are in Additional File 3.

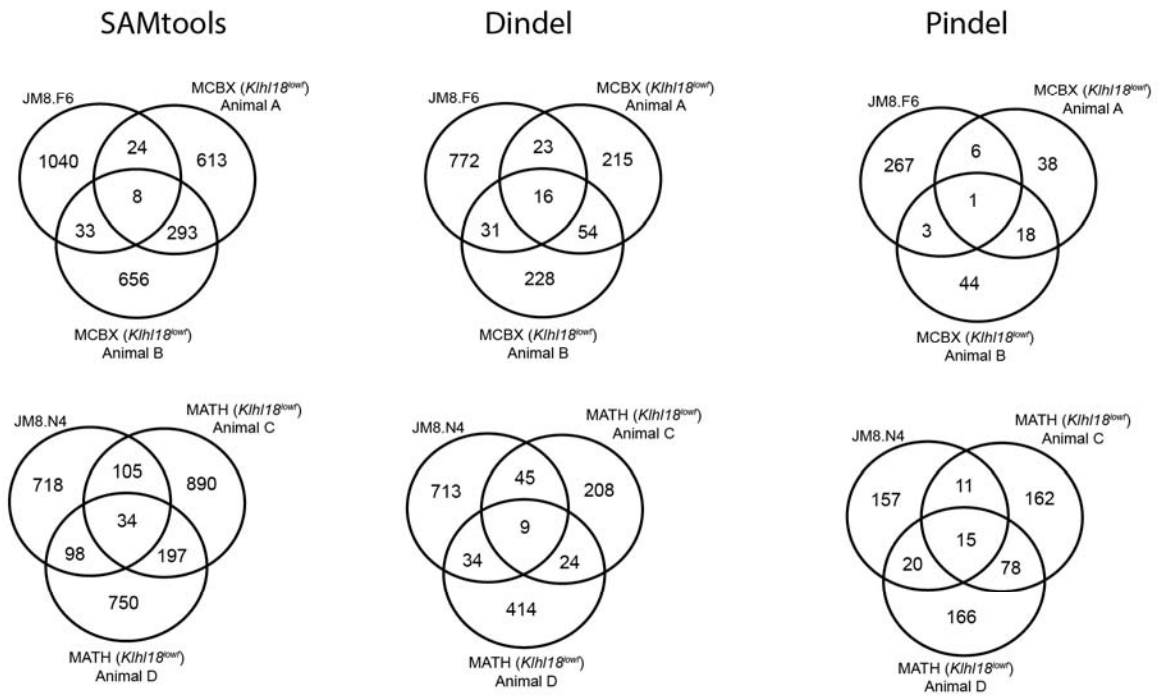

Fig. S7. A subset of variants identified by each caller were passed on from parental ES cells to mice created using that ES cell line.

Venn diagrams showing the number of high quality variants in two ES cell lines (JM8.F6 and JM8.N4) which were shared by mice from lines created using those ES cell lines (MCBX and MATH), detected by SAMtools, Dindel and Pindel. Both these mouse lines also carry the spontaneous *Klhl18^{lowf}* mutation, which was not seen in any of the ES cell lines. None of the variants identified by BreakDancer were shared between the ES cells and the mice (Additional File 2: Table S4).

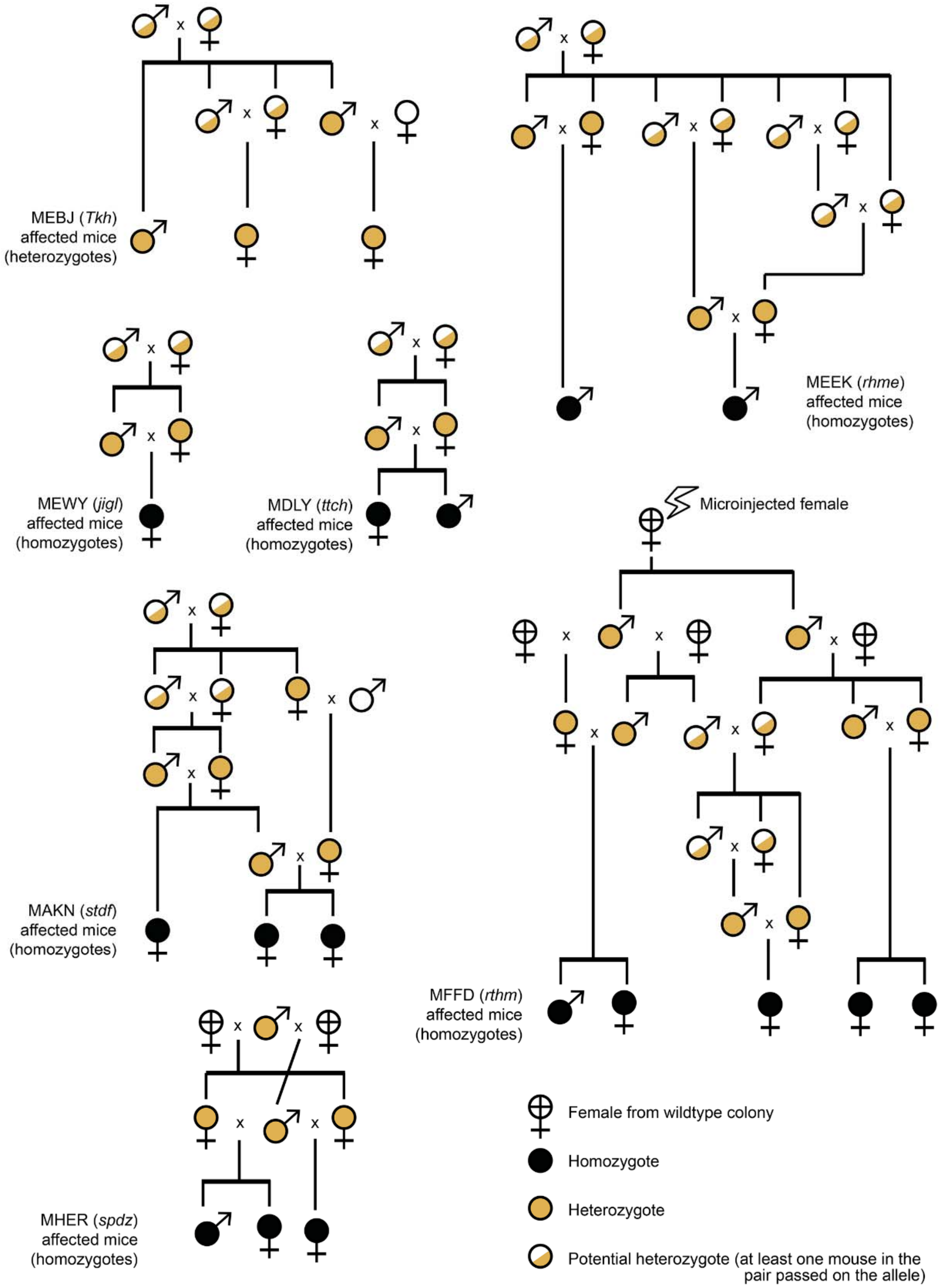

Fig. S8. Pedigrees of the affected mice showing the latest possible point at which each mutation could have arisen.

Pedigrees of mouse lines for seven of the eight mutations described here. The *Klhl18*^{lowf} allele is thought to have arisen in the wildtype colony and is not shown. For each of the other lines, the pedigree shows the latest possible point at which the mutation could have arisen. Homozygotes are shown in solid black circles, heterozygotes (obligate carriers for the recessive mutations or mice carrying the semidominant *Atp2b2*^{Tkh} allele) are shown in yellow, and where either or both mice in a pair may have been carrying the mutant allele, this is indicated by the white/yellow colour. In only one line (MFFD, *rthm*) does the mutation definitely originate at or before the point of microinjection of the targeted ES cells. Only the mice at the bottom of each pedigree, marked by the text, are known to have been affected (and therefore homozygotes or, for *Atp2b2*^{Tkh}, heterozygotes); all the other genotypes and phenotypes are inferred.

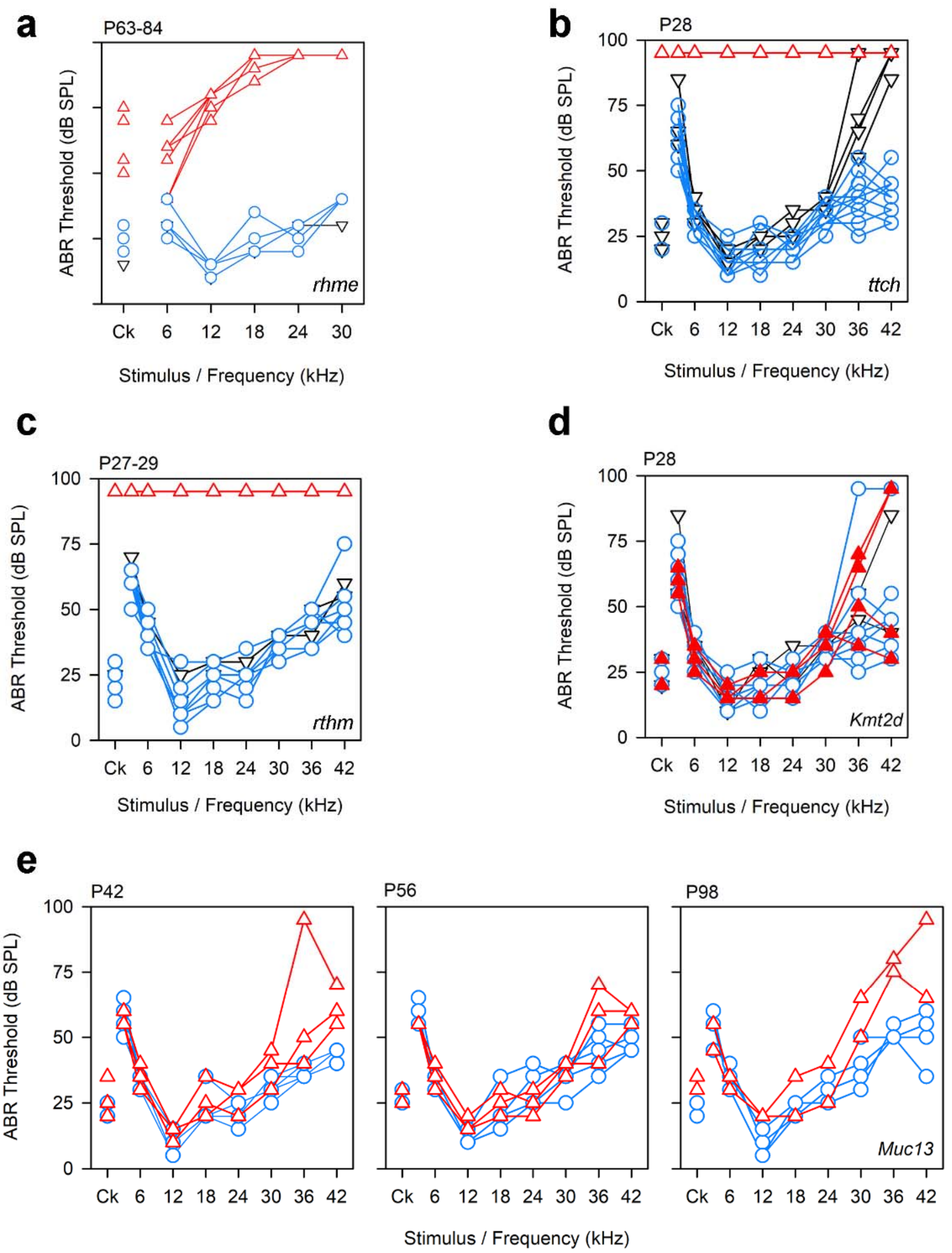

Fig. S9. Individual ABR thresholds.

Individual ABR thresholds plotted where fewer than 6 mice in a single group were tested. **a** Individual ABR thresholds for mice wildtype (n=1, black inverted triangles), heterozygous (n=5, blue circles) and homozygous (n=6, red triangles) for the *rhme* deletion (MEEK colony) at P63-84. Mean thresholds are shown in Fig. 4b. **b** Individual ABR thresholds from P28 mice homozygous (n=6, red triangles), heterozygous (n=10, blue circles) and wildtype (n=4, black inverted triangles) for the *Tbx1*^{ttch} allele (MDLY colony), p.D212N. Mean thresholds are shown in Fig. 5a. **c** Individual ABR thresholds of mice homozygous (n=6, red triangles), heterozygous (n=7, blue circles) and wildtype (n=2, black inverted triangles) for the *rthm* allele (MFFD colony) at P28±1 day. Mean thresholds are shown in Fig. 7a. **d** Individual ABR thresholds for P28 mice homozygous (n=4, red triangles), heterozygous (n=8, blue circles) or wildtype (n=2, black inverted triangles) for the *Kmt2d* deletion (MDLY colony). Mean thresholds are shown in Additional File 1: Fig S6b. **e** Individual ABR thresholds for mice homozygous (red triangles) or heterozygous (blue circles) for the *Muc13* mutation (MDLY colony) at P42, P56 (n=5 heterozygotes, 3 homozygotes) and P98 (n=4 heterozygotes, 2 homozygotes). All these mice were heterozygous or wildtype for the *Tbx1*^{ttch} allele. Mean thresholds are shown in Additional File 1: Fig S6g. Data underlying these plots are in Additional File 3.
